# Magmatic origin of the Dewar magnetic anomaly: Implications for an early lunar dynamo

**DOI:** 10.1126/sciadv.aec0341

**Published:** 2026-09-23

**Authors:** Xi Yang, Anna Mittelholz, Adrien Broquet, Max Moorkamp

**Affiliations:** ^1^ Department of Earth and Planetary Sciences, ETH Zurich, Zurich, Switzerland.; ^2^ Institute of Space Research, DLR, Berlin, Germany.; ^3^ Institute of Applied Geosciences, TU Berlin, Berlin, Germany.

## Abstract

The Moon’s ancient magnetic field provides critical insights into lunar thermal and magnetic evolution, yet the lifetime of the interior dynamo remains debated. Returned samples yield complex and contradictory paleomagnetic records, while orbital data reveal crustal magnetic anomalies of uncertain origin from either a core dynamo or transient impact-generated fields. Here, we jointly invert gravity and magnetic observations in the region around the Dewar swirl, a high-albedo feature associated with the Dewar magnetic anomaly. We identify a shallow, magnetized, high-density body consistent with buried mare basalt. Our estimates require a paleointensity exceeding 11 microtesla, suggesting an active lunar dynamo about 4.2 billion years ago, as constrained by the superposed basin ejecta. The results also show that swirl formation requires horizontal magnetization and iron oxide enrichment. These findings link a magnetic anomaly to its geologic origin and magnetizing field, providing constraints on the lunar magnetic and volcanic history.

## INTRODUCTION

The Moon is thought to have once hosted a dynamo that produced a global magnetic field, which is supported by the paleomagnetic record of the returned lunar samples and the presence of a magnetized crust observed from orbit (*1*). The thermal requirements to power a core dynamo provide important information on the timing of differentiation and early geodynamic history. Remanent magnetization preserved in the crust provides a record of crustal formation and modification events, such as impact basin formation or volcanic emplacement, in the presence of an ancient field (*2*). Such processes result in localized magnetic anomalies that are observable today. Furthermore, interactions between these anomalies and the solar wind modify the local plasma environment, deflect solar wind ions, and affect space weathering on the lunar surface materials (*3*). This process has potential implications for the safety of future robotic or human lunar exploration. Therefore, the study of lunar magnetism has major implications for the interior structure and thermal history, the physics of planetary dynamos, and future exploration missions.

**Fig. 1.**
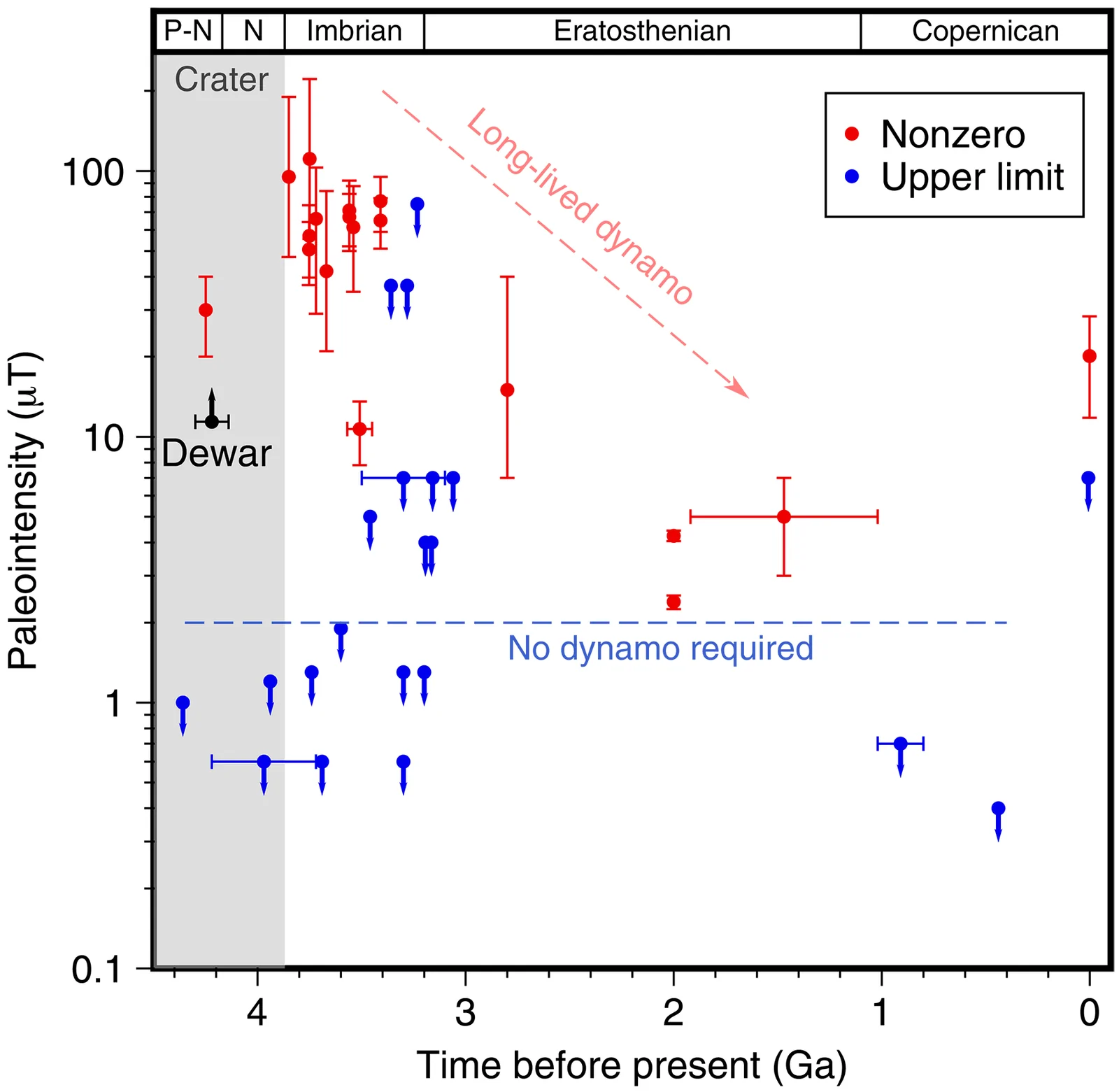
Paleointensity summary. The paleointensity results from paleomagnetic analyses (*5*–*20*). Nonzero measurements (red) indicate an active dynamo with the trend of the surface paleointensity of the long-lived dynamo shown by the red dashed arrow. Others present upper limits of a possible dynamo field (blue). The blue dashed line highlights the paleointensity of 2 μT, and results below this illustrative limit have been interpreted as evidence for the absence of a lunar dynamo (*18*–*20*). An active lunar dynamo before about 3.85 Ga from the cratering record is shown in gray (*25*). The estimated minimum paleointensity from this study is shown in black. An age error bar is shown only when the uncertainty is 0.05 Ga.

The current records of the lunar dynamo come from paleomagnetic studies of samples returned from the Moon and observations of crater magnetization from orbiters (Fig. 1). Many paleomagnetic analyses of lunar samples suggest that a long-lived lunar dynamo operated from at least about 4.25 billion years ago (Ga) to about 3.5 Ga with a field strength similar to that of the present Earth (*4*–*10*). The intensity of the dynamo field then weakened drastically and was sustained to sometime between about 1.92 and 0.8 Ga (*11*–*18*). However, recent paleomagnetic analyses have led to conflicting results and, instead, suggest the absence of a dynamo field from about 4.36 to 3.2 Ga (*19*, *20*), limiting its activity to the earliest period of lunar history and even questioning whether a lunar dynamo ever existed. Some ancient samples might have recorded transient impact-generated fields (*21*), similar to what has been proposed to explain the youngest (a few million years old) high-paleointensity records. In parallel, the magnetic signatures of lunar craters observed from orbit have been proposed as evidence of a dynamo before the beginning of the Imbrian period at ∼3.85 Ga (*22*–*25*). Therefore, to resolve ongoing debates regarding the timing and longevity of the lunar dynamo, additional paleointensity records, particularly for early periods from about 4.3 to 3.8 Ga with clear evidence for or against a dynamo, are required.

**Fig. 2.**
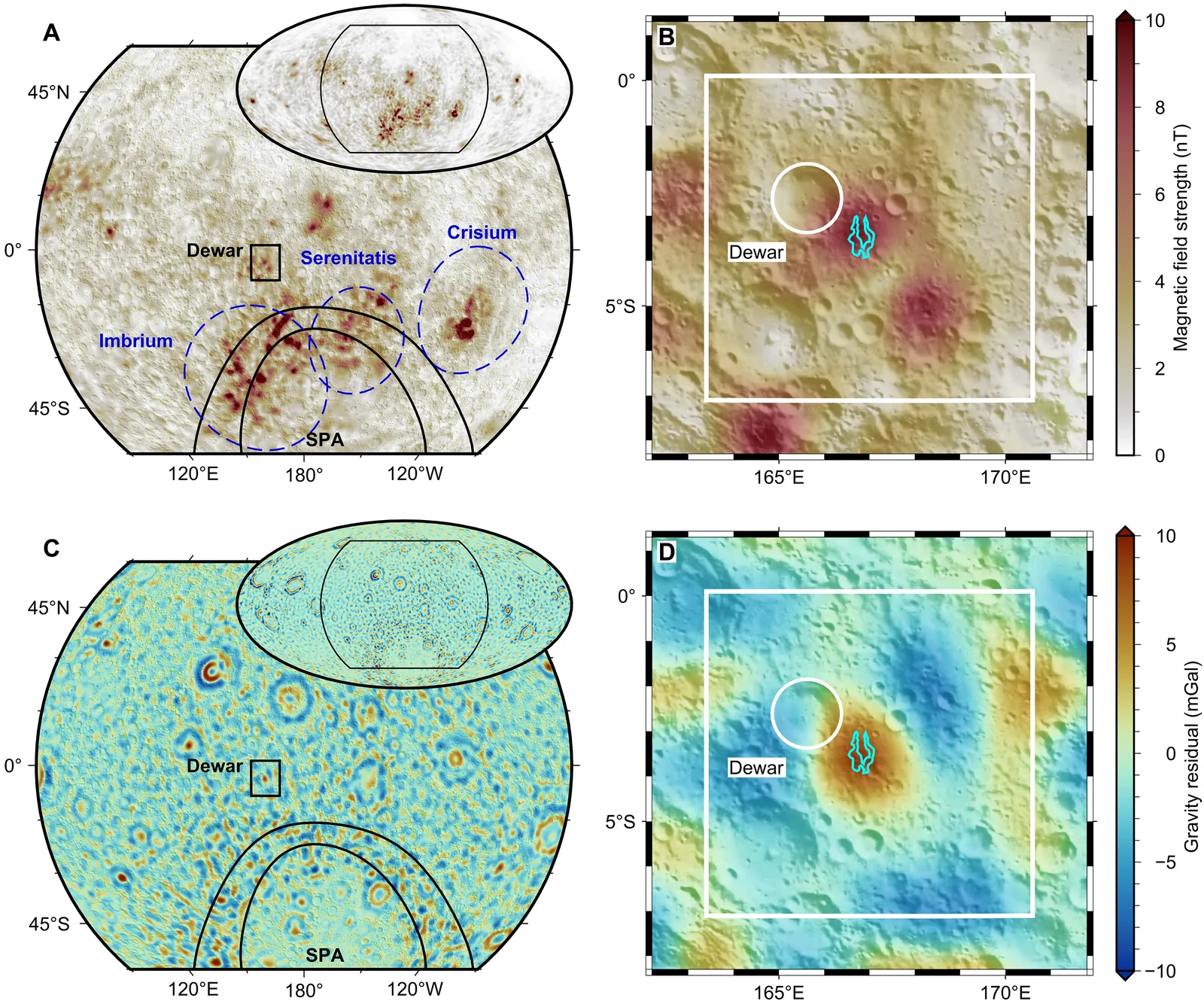
Magnetic field strength and gravity residual at 30 km altitude. (**A** and **B**) The magnetic field strength from (*27*); (**C** and **D**) the gravity residual obtained by removing signals associated with the surface and Moho reliefs. In (A), the dashed blue circles denote the antipodes of the Imbrium, Serenitatis, and Crisium basins with radii from (*61*). [(A) and (C)] The solid ellipses denote the inner floor and outer rim of the South Pole–Aitken (SPA) basin (*83*). (B) and (D) correspond to the black box in (A) and (C) highlighting the region around the Dewar swirl [cyan outline from (*35*)] and the Dewar crater rim (white circle). The solid white boxes show the region displayed in Fig. 3. Maps are shown in [(A) and (C)] the Mollweide equal-area centered on the lunar farside and [(B) and (D)] Mercator projection, respectively.

The lunar crust preserves a geophysical record of thermal, magmatic, and impact-related processes after the initial differentiation of the Moon (*26*). Through these modifications, a portion of the lunar crust acquired remanent magnetization (Fig. 2A), leading to pronounced and heterogeneously distributed magnetic anomalies detectable from orbit (*27*–*30*). On a global scale, magnetic anomalies are concentrated on the farside southern hemisphere (Fig. 2A). This could be the result of magnetized iron-rich materials from the South Pole–Aitken (SPA) impact event (*31*). Alternatively, it could be related to the acquisition of shock remanent magnetization in the presence of the impact-amplified magnetic field near the antipodes of the Imbrium, Serenitatis, and Crisium (*32*, *33*). On a regional scale, the formation mechanism responsible for many strong anomalies remains unknown. Some anomalies are associated with large impact basins, supporting the hypothesis of thermoremanent magnetization of the impact melt (*22*–*24*). However, only a small fraction of lunar craters exhibit magnetic anomalies (*25*), indicating that impact-related mechanisms alone cannot explain the full range of observed features. Nonimpact origins, such as magmatic emplacement or intrusive bodies, have also been proposed (*34*), but these are more difficult to investigate due to the limited ability to resolve subsurface structures.

One set of geological features that may help investigate the nonimpact origins of lunar crustal anomalies is lunar swirls, which are high-albedo sinuous surface features that consistently coincide with magnetic anomalies (*35*, *36*). Swirls have been proposed to be shaped by deflection of the solar wind in regions of crustal magnetic fields (*36*–*39*) or by transportation of fine-grain dust (*40*–*42*). Both models provide a connection between the morphology of the swirl and the structure of the crustal magnetic field [for example, (*43*)]. Therefore, analysis of swirls can provide insight into the geologic processes that have shaped the crust. The best studied example, Reiner Gamma, has been proposed to be the result of magnetic fields associated with an impact melt sheet deposit (*44*), distal ejecta of the Imbrian basin (*29*), or an intrusive magmatic dike (*34*). Investigations have associated Reiner Gamma with a subsurface magnetized structure connected to the Marius Hills volcanic complex (*45*, *46*), suggesting a possible magmatic origin.

If a magnetic anomaly originates from a geologic process that also generates a density anomaly (e.g., magmatic processes), a correlation between observed gravity and magnetic data is expected. Although dominant structures such as large-scale linear gravity anomalies (*47*) and linear magnetic anomalies (*48*) do not show a correlation, some gravity and magnetic field correlations have been observed regionally (*49*). In such regions, combined analysis of these datasets is well suited to constrain the structure and formation history of the crust. One such region was identified in the vicinity of the Dewar swirl, a single isolated swirl approximately 25 km across (*35*). The Dewar swirl is located northwest of the SPA basin in the farside highlands near the equator, next to the Dewar crater, and is associated with strong gravity and magnetic anomalies (Fig. 2). In addition, the Dewar region displays a prominent surface geochemical anomaly with an elevated concentration of iron, titanium (as shown below), thorium, and pyroxene, which has been interpreted as a buried mare deposit, also called cryptomare (*50*, *51*).

Our study analyzes the crustal structure of the Dewar region to provide insights into the origin of crustal magnetic anomalies and lunar swirls. We conduct the first joint inversion of lunar gravity and magnetic field data and characterize the density and magnetization structure of the crust beneath the Dewar swirl. Then, we combine the inversion results, surface topography, and compositional data to investigate the origin of the Dewar swirl and the associated magnetized body. Last, we estimate the minimum paleointensity that is required to generate the density and magnetization anomaly associated with the Dewar swirl based on the inverted model and discuss the implications of our results for the lunar dynamo.

## RESULTS

This study uses gravity residual and magnetic field data (fig. S1) to resolve the three-dimensional (3D) crustal structure of density and magnetization simultaneously (see Methods). The gravity residual is derived from the GRGM1200B gravity field model (*52*) by subtracting gravity signals from the surface topography (i.e., Bouguer correction) and the modeled Moho relief using the DSP model (*53*). The model calculates a finite-amplitude Bouguer correction with an assumed density of 2550 kg m^−3^ and inverts the associated Bouguer anomaly for a 40-km-deep Moho relief with an assumed density contrast of 670 kg m^−3^ following previous work (*54*). Some residual short-wavelength gravity anomalies cannot be explained by surface topography or Moho relief, and these are expected to highlight density anomalies in the crust (*55*, *56*). The magnetic field data are derived from the lunar magnetic field model from (*27*). The maximum spherical harmonic degrees used in our analyses of the gravity residual and magnetic data are 550 and 450, respectively. To better resolve the small-scale features while preserving the dominant large-scale features, we sample the gravity and magnetic data at 6 km altitude to represent near-surface observations and 30 km altitude to approximate satellite measurements.

Resolving the crustal structure using inversions of potential field data is inherently nonunique. As gravity and magnetic data sense the structure differently, combined analysis can alleviate this issue and allow simultaneous constraints on the subsurface density and magnetization. The goal of a joint inversion is to find whether the density and magnetic anomalies originate from the same geological structure. If no such solution exists, it is impossible to fit both observed gravity and magnetic datasets while simultaneously fulfilling the imposed constraints, and we can refute the hypothesis of a common origin.

Our joint inversion of gravity and magnetic data applies an objective function composed of misfit, regularization, and coupling terms (*55*, *57*, *58*). The coupling terms favor models in which density anomalies and magnetization are spatially collocated. A large coupling weight enforces strongly collocated structures, while a low coupling weight allows the model to better fit observations and results in smoother models due to regularization. Our inversion aims for the best coupled model parameters that also fit the observations. Therefore, the inversion begins with a strong coupling constraint between gravity and magnetic field anomalies. Such a high weight mandates that every gravity and magnetic anomaly in the investigated region originates from collocated sources, leading to a poor fit to the observations for noncollocated sources. Thus, we iteratively relax the coupling weight to reduce the coupling requirement (fig. S2). This approach allows us to dissociate the gravity and magnetic sources that do not originate from the same location, while preserving collocation where observations imply a common source.

**Fig. 3.**
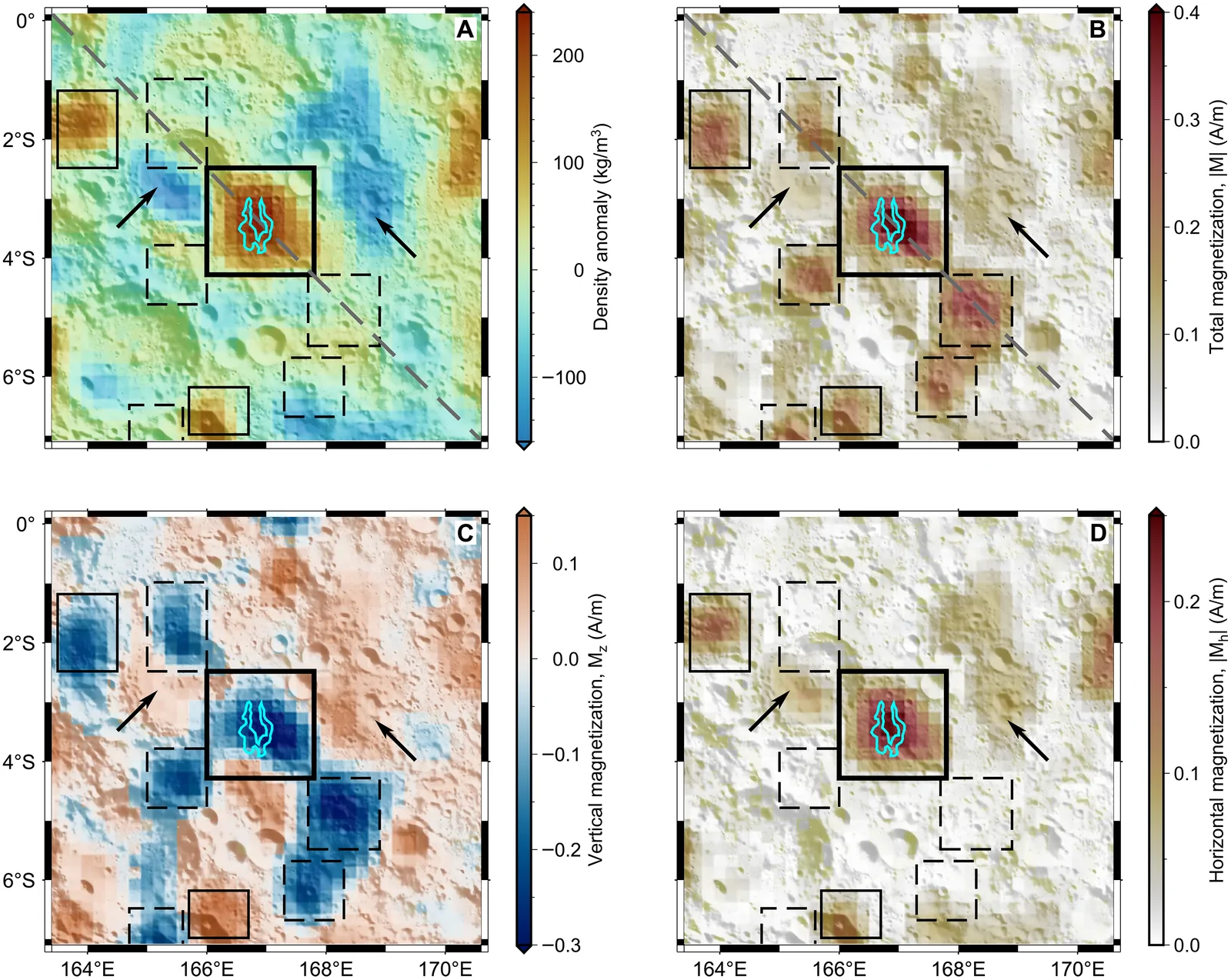
Magnetization and density surrounding the Dewar swirl at 1-km depth. (**A**) Density, (**B**) total, (**C**) vertical, and (**D**) horizontal magnetization, where the positive direction of vertical magnetization is downward. The solid and dashed boxes denote magnetized bodies associated with a positive and lacking density anomaly, respectively. The anomaly associated with the Dewar swirl (cyan) is highlighted by the bold box. Arrows point to additional magnetized bodies with negative density anomaly. The gray dashed lines in the upper row denote the profiles shown in Fig. 4 (A and B).

The resulting 3D crustal model of density and magnetization reveals several magnetized bodies in the region surrounding the Dewar swirl, some of which are associated with a density anomaly (Fig. 3). All magnetized bodies with a positive density anomaly consistently exhibit horizontal magnetization, suggesting dipping magnetization relative to the vertical. Among those, the Dewar swirl is associated with the strongest anomaly body (hereafter named the Dewar anomaly) with a maximum magnetization of about 0.4 A/m and a density about 250 kg/m^3^ higher than the surrounding crust. In contrast, magnetized bodies that lack an associated density anomaly uniformly exhibit no horizontal magnetization and a negative vertical magnetization of similar strength to that observed in other magnetized anomalies. Last, two weakly magnetized bodies are found to be associated with weak negative density anomalies. All of these anomalies extend down to a depth of about 10 km (fig. S3).

**Fig. 4.**
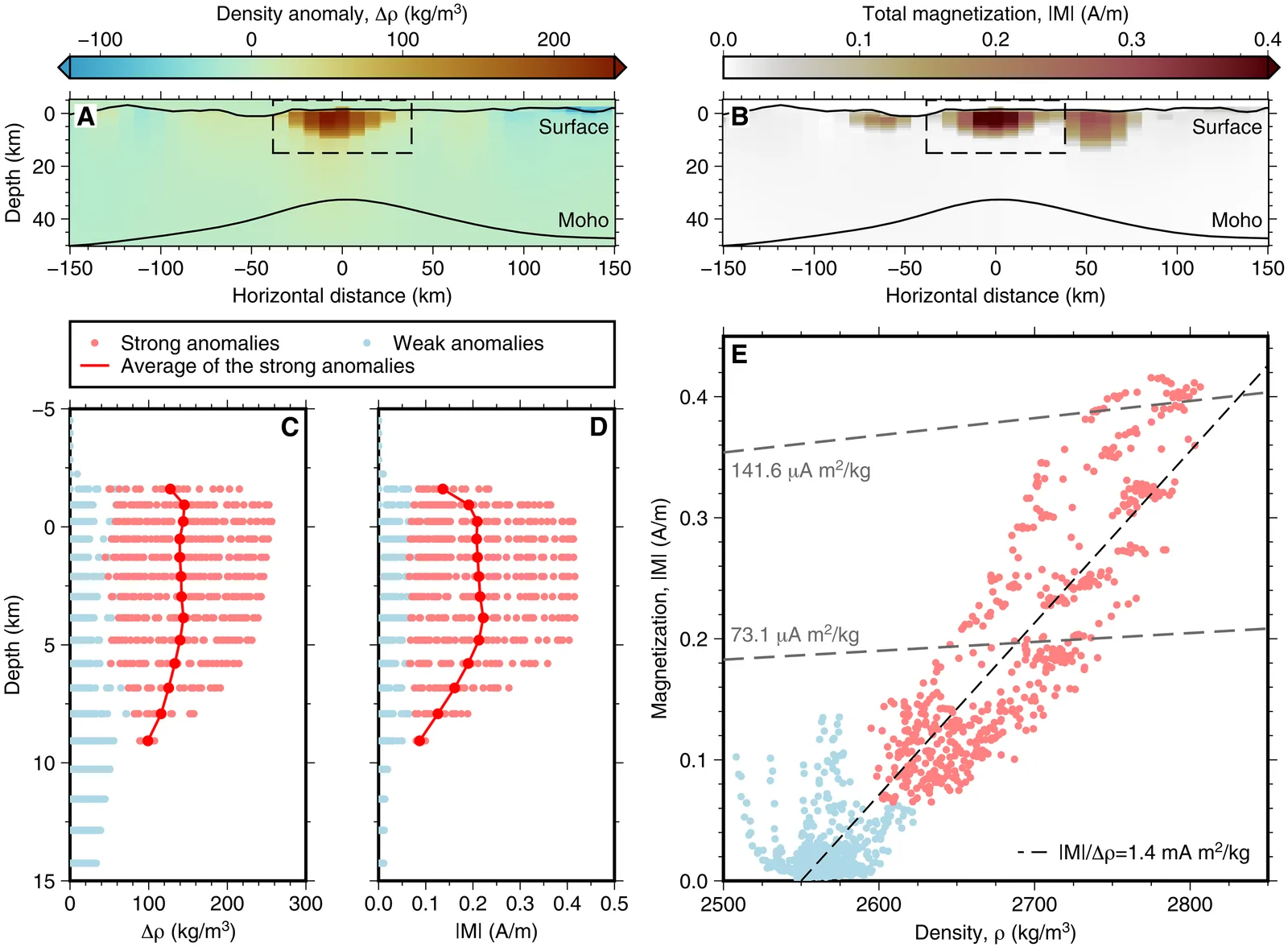
Density and magnetization of the Dewar anomaly. (**A** and **B**) The vertical profiles of the (A) density anomaly and (B) total magnetization across the Dewar anomaly. (**C** to **E**) Only the region below the Dewar swirl [dashed box in (A) and (B) and bold box in Fig. 3) at depths <15 km is shown. [(C) and (D)] The distribution of the (C) density anomaly and (D) magnetization of the Dewar anomaly as a function of depth. For visual support, weak (blue) and strong (red) parts are separated, where strong is defined as having density and magnetization that are both larger than the 72.5th percentile within this region. (E) Density is represented by the sum of density anomalies in (A) and the crustal density of 2550 kg/m^3^ (see Methods). The black dashed line denotes the average magnetization-to-density anomaly ratio. The average and the 95th percentile of the magnetization-to-density ratio of strong anomalies are shown by the dashed gray lines, respectively.

Our inversion results suggest that the density and magnetization structure of the Dewar anomaly originates from one source body of about 60 km width and extending down to about 9 km depth (Fig. 4, A and B). To better characterize the Dewar anomaly, we extract cells where both density and magnetization exceed the 72.5th percentile within the region around the swirl (box in Fig. 3) at depths of <15 km (Fig. 4, C to E). This prominent part of the strong anomaly extends from the surface to about 5 km depth (Fig. 4, C and D). Here, the average values reach about 150 kg/m^3^ for density and 0.2 A/m for magnetization, respectively. Notably, the density and magnetization of the strong anomaly show a pronounced linear correlation (Fig. 4E), with an average magnetization per anomaly density of 1.4 mA m^2^/kg. In contrast, the low density and magnetization parts lack correlation.

Although improved by the joint approach, potential field inversions have limited ability to resolve the deep structure of the crust (∼30 km; fig. S4) because of the nonuniqueness in acceptable solutions. Synthetic tests suggest that our inversion can well resolve the shallow magnetized bodies and the horizontal extent of density structure, but it always allows for the coupling solution regardless of whether the anomalies are indeed overlapped vertically (fig. S5). Therefore, the vertical density structure should be interpreted with caution. Nevertheless, our results show that Dewar gravity and magnetic anomalies originate from the same source body, which is valid within the uncertainties of the data. However, we also cannot discard all other alternatives, as is typical for gravity or magnetic field inversions.

To support our inferred shallow origin for the magnetic and gravity anomaly, we conduct a degree-depth investigation (*59*). In a Bouguer anomaly map, low-degree components tend to be associated with large-scale and deep structures, while high-degree components are associated with small-scale and shallow structures. By filtering out low-degree components, the resulting Bouguer anomaly maps highlight shallow subsurface density features. This approach independently suggests the presence of a large-scale structure with depths exceeding about 42 km and a shallow small-scale structure within less than about 11 km below the Dewar swirl (fig. S6). These depths are consistent with the Moho relief depth estimated from gravity and topography, as well as with shallow density anomalies, confirming our inversion results (Fig. 4A).

## DISCUSSION

**Fig. 5.**
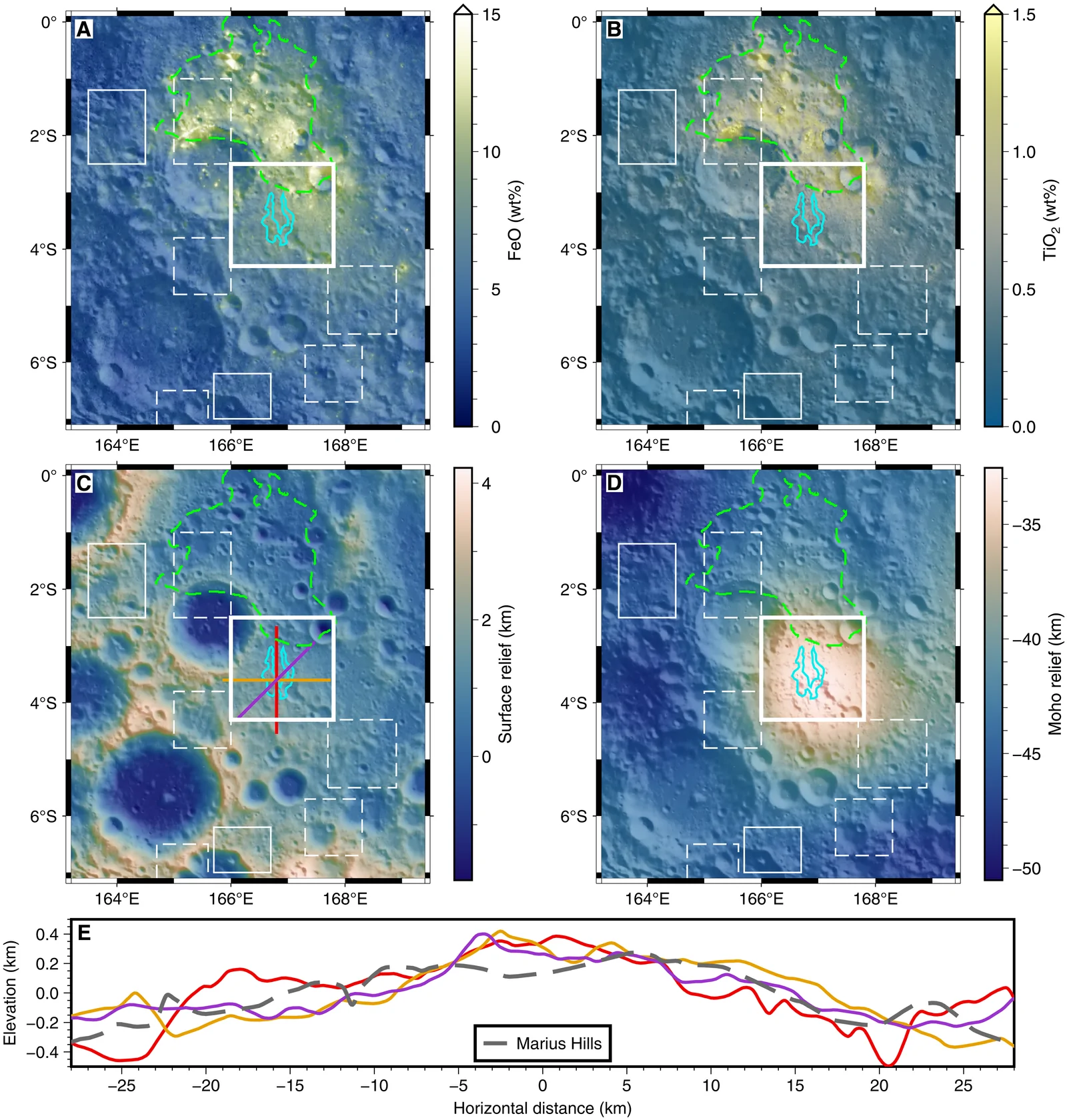
Maps of the region surrounding the Dewar swirl. Surface abundance of (**A**) FeO and (**B**) TiO_2_, (**C**) topography, and (**D**) Moho relief. White boxes denote the locations of magnetic anomalies as in Fig. 3. The Dewar swirl is highlighted in cyan, and the Dewar cryptomare region (*51*) is highlighted by the dashed green outline. The surface FeO and TiO_2_ abundance map was derived from the Clementine UVVIS datasets (*84*, *85*). Colored lines correspond to (**E**) topographic profiles with ejecta material subtracted starting from south or west (for details, see fig. S7). The topographic profile of Marius Hills along the East-West direction centered at 13.27°N, 51.96°W is shown in dashed gray for comparison.

The resulting crustal structure surrounding the Dewar swirl provides clues for the mechanisms of swirl formation. Two key factors might be required to explain the presence of the swirl: sufficiently strong horizontal magnetization (Fig. 3D) and FeO-rich surface material (Fig. 5A). This combination is consistent with the hypothesis that local magnetic fields shield the surface from solar wind particles; while horizontal fields deflect charged particles, vertical fields channel them toward the planet. The resulting shielding locally reduces space weathering, thereby preserving a higher surface albedo. However, because not all horizontally magnetized anomalies produce swirls, this condition alone is not sufficient. The Dewar swirl lies within a geochemically anomalous region rich in iron oxide (FeO), whereas none of the other magnetized bodies with horizontal magnetization share this surface geochemistry. This observation supports global trends, indicating that lunar swirls preferentially occur in regions with a high FeO content (*35*). We propose that space weathering of FeO-rich material, which generates opaque submicroscopic iron, lowering the surface albedo, is required to lead to observed brightness contrasts. Thus, only FeO-rich regions that are effectively shielded from space weathering can form an albedo high in contrast to their surroundings, resulting in swirls.

The positive density anomaly of the Dewar anomaly can be explained by intrusive basaltic materials. This intrusive body could be associated with a near-surface cryptomare, which has been identified in the investigated region (*50*, *51*). The inference of cryptomare itself is supported by buried extrusive low-Ti basalt with elevated FeO (Fig. 5A), TiO_2_ (Fig. 5B), and mantle-derived materials at the surface (*51*). After removing the impact ejecta and background topographic trend (fig. S7), we detect a mound-like topography above the Dewar anomaly that is comparable to the Marius Hills volcanic complex (Fig. 5, C and E). Magmatic intrusion–induced uplift is widely observed on the Moon, including in floor-fractured craters (*60*). Thus, our observation supports the presence of an intrusive body, such as a buried volcano, under the Dewar swirl. Last, the Dewar anomaly displays the thinnest regional crust (Fig. 5D), which is a preferable condition for the extrusion and eruption of magma. All of these observations are consistent with the magmatic origin of the Dewar anomaly.

A magnetic body will acquire magnetization if it is emplaced in the presence of a dynamo field, and we observe several magnetic anomalies. The presence or absence of a collocated density anomaly might indicate different formation mechanisms of such a body. Magnetized bodies other than Dewar that exhibit positive density anomalies lack association with geochemical anomalies and are not located near the inferred cryptomare (Fig. 5, A to D). For these, associated density anomalies are consistent with intrusive dikes or buried local emplacements of impact melt. Magnetized bodies with no density anomaly exhibit only negative vertical magnetization components with similar strength (Fig. 3A), suggesting a common formation period and mechanism. Such anomalies might originate from distal ejected materials from large impact events, such as the SPA basin (*31*). In contrast, weakly magnetized bodies accompanied by negative density anomalies may have formed through impact shock from smaller regional impacts. Such a process could increase crustal porosity, manifesting in a negative density anomaly, while simultaneously generating a shock remanent magnetization. However, this process remains poorly understood, and whether it can generate a detectable magnetic anomaly is still unclear [for example, (*25*)]. Generally, any magnetic anomalies observed in the vicinity of the Dewar swirl are unlikely to result from antipodal shock magnetizing effects (*32*, *33*), as the studied region is located outside the antipodes of large impact structures (*61*).

To further assess the possible magmatic origin of the Dewar anomaly, we compare our model results with geochemical constraints on magnetization formation. If the observed magnetization results from the metallic iron enrichment produced by the reduction of ilmenite during slow basalt cooling (*62*) in the presence of a lunar dynamo, the density anomaly constrains the volume of basalt and thus the amount of available ilmenite. Assuming a paleointensity of 70 μT, we estimate that ∼31% of ilmenite would be reduced to iron (fig. S8, Methods), consistent with the timescales expected for intrusive basalt cooling.

The intensity of the magnetic field at the time of the formation of the Dewar anomaly is unknown. Yet, as the availability of magnetic material to form the anomaly is limited, our inferred magnetization and density can be used to provide constraints on the paleointensity. If the anomaly body contains the highest possible concentration of iron, a minimum magnetic intensity is needed to generate the observed magnetic anomaly. This means that the lower bound of the paleointensity during the formation of the Dewar magnetic anomaly can be estimated. We find that the minimum paleointensity is proportional to the crustal magnetization per mass. For the derived average value of the Dewar anomaly, 73.1 μA m^2^/kg (Fig. 3E), the estimated minimum paleointensity is about 11.4 μT, and 22.0 μT if we use the largest magnetization per mass of 141.6 μA m^2^/kg. These estimates are consistent with the surface intensity of tens of microtesla that support the presence of a lunar dynamo (Fig. 1), indicating that the Dewar anomaly was magnetized by a dynamo field during its slow cooling. We note that our minimum constraint assumes a stable field during the cooling timescale of tens of millions of years for a magmatic body with a size similar to the Dewar swirl. Any instabilities would reduce net magnetization and require an increased paleomagnetic field (*63*, *64*). An alternative explanation for the magnetizing field is the transient impact-generated fields, which is unlikely because such events could only thermally magnetize a very small portion of the intrusive body within their short duration of only a few hours (*33*).

The formation period of the Dewar anomaly is crucial for reconstructing the history of the lunar dynamo. The Nectarian Dewar crater postdates the cryptomare (*50*), suggesting that the formation of the Dewar anomaly must have occurred before the end of the Nectarian period at about 3.85 Ga. Furthermore, a rather precise constraint comes from the impact ejecta material that covers the Dewar cryptomare. The Dewar anomaly should have formed later than the SPA basin. Otherwise, the Dewar region would be expected to be covered by a thick ejecta blanket of a few kilometers (*65*) without distinct surface chemical anomalies. Besides, Dewar cryptomare should have formed before the nearest Freundlich-Sharanov (FS) basin because the cryptomare is superposed by its ejecta (*51*). The model ages of the SPA and FS basins are about 4.31 and 4.14 Ga, respectively (*66*). Therefore, we conclude that the Dewar anomaly formed during the pre-Nectarian period at about 4.22 ± 0.08 Ga (Fig. 1). This is consistent with the magnetic signatures of the crater (*25*) and a subset of paleomagnetic results. The estimated minimum paleointensity of >11 μT (>22 μT to explain the strongest magnetization) is consistent with the paleomagnetic analysis of the 4.25 Ga lunar troctolite (*5*), the earliest nonzero paleomagnetic estimate. The null results of the paleomagnetic analysis could be explained by an episodic dynamo or a dynamo that frequently reversed its polarity, consistent with the coexistence of magnetized and demagnetized craters of similar ages (*25*).

Our results have key implications for the lunar dynamo mechanism, which must have been capable of generating and sustaining a magnetic field strong enough to account for observed paleointensity, despite the small lunar core. This challenge has been presented in the context of paleomagnetic studies arguing for a dynamo field. The estimated paleointensity of >11 μT at about 4.2 Ga cannot be explained by thermal convection (*67*, *68*), core crystallization (*69*, *70*), impact-induced nonsynchronous rotation (*71*), and inner core precession driving viscous friction (*72*); these mechanisms only generate surface intensities of a few microtesla. Thermal convection in a basal magma ocean (*73*) and differential precession of the mantle and core (*74*) could generate surface intensities of a few tens of microtesla consistent with our estimates. However, it has been questioned whether a basal magma ocean is sufficiently conductive and whether mantle precession could generate a stable dynamo (*2*). Enhanced cooling during the formation of Ti-rich material (*75*) could generate a surface intensity of >50 μT for several centuries, but its efficiency in the early period of about 4.2 Ga is unclear. Perhaps the easiest explanation for the observed paleointensity is a combination of different dynamo mechanisms mentioned above.

Future investigations of lunar magnetism and crustal structure will be essential to address the limitations of this study. First, owing to the altitude of satellite observations, our model cannot resolve the fine-scale magnetization comparable to the albedo transition length scales of lunar swirls. Second, inversion of the gravity field cannot resolve the possible magmatic pathways connecting the shallow magma chamber and the crust-mantle boundary. This could be addressed with complementary geophysical observations, such as seismic data. Third, current paleomagnetic constraints on dynamo field intensity are derived primarily from Apollo nearside samples; a more comprehensive assessment will require samples from farside highlands. Upcoming missions such as Lunar Vertex, Chang’e 7 and 8, and Artemis may provide the necessary observations and samples. While resolving these aspects remains a key objective for future studies, our results demonstrate that orbital data alone can place constraints on the lunar dynamo that are independent of the sample records. The methodology developed in this study offers particular promise for investigating planetary bodies in which direct sampling is sparse or absent.

## METHODS

### Gravity data processing

To focus on the density of the crust, we use what we call the gravity residual, which is estimated as follows. We expand the spherical harmonic model GRGM1200B of the lunar gravity potential (*52*) to a maximum degree of 550. From the predicted free-air gravity, we subtract gravity signals from the surface topography (Bouguer correction) using the LOLA shape model and from the crust-mantle interface. The gravitational signature of the crust-mantle interface is modeled using the DSP package (*53*) assuming an average crustal thickness of 40 km, a crustal density of 2550 kg/m^3^, and a mantle density of 3220 kg/m^3^ [the average values from (*54*)]. A half-degree 80 downward continuation filter is applied to the topography-corrected gravity field before solving for the Moho relief. Filters with smaller wavelengths lead to unrealistic oscillations of the Moho (*54*), and filters with a longer wavelength erase the expected crustal signature of impact basins. The gravity residuals can thus be understood as Bouguer anomaly signals arising from unrealistic oscillations of the crust-mantle interface and therefore must have their source within the crust. We attribute these residuals to variations in crustal density related to composition or porosity. We note that portions of the long-wavelength gravity signals might correspond to large-scale density anomalies in the crust, which cannot be uniquely resolved.

### Joint inversion

For our joint inversion, we assign an uncertainty of 1 mGal to the gravity data based on the predicted data error at the surface of the model GRGM1200B (*52*). This is a conventional setup, as the truncation of the model and the upward continuation may decrease the uncertainty. Any further decrease in the uncertainties does not affect the conclusions of our study. For the magnetic data, because no estimates of uncertainties are provided for the input model, we assign an uncertainty of 0.1 nT or 2% of the value, whichever is greater. Solar wind–generated fields or instrument noise might lead to contamination of individual orbital tracks, but they are expected to be small for a model that incorporates multiple repeat orbits, such as the model by Tsunakawa *et al.* (*27*). Hence, the small uncertainty assumes that the crustal field provides a very good description of the field. In addition, to confirm the consistency of the predicted magnetic field structure, we use data generated from a second magnetic field model (*28*). Note that when using this model for the inversion, the results are found to be similar to those shown in Fig. 3, and the estimate of paleointensity only decreases by less than 10%, which does not affect the main conclusions of this work.

We use the jif3D joint inversion framework (*76*) with a recently developed coupling constraint based on the variation of information (*58*) and jointly invert for the density and magnetization structure in the Dewar region. Variation of information (VI) is a measure of the amount of information that one variable carries about another variable. This methodology was recently proposed for terrestrial (*57*) and subsequently Martian (*55*) investigations.

The joint inversion approach solves a nonlinear optimization problem to minimize an objective function. The objective function (Φ) is composed of the misfit terms of the data for the gravity residual $\Phi_{d,\text{grav}}$ and the components of the magnetic field data $\Phi_{d,B_{i}}$, (where *i* represents the components *x*, *y*, and *z* in Cartesian coordinates), the L2 regularization terms $\Phi_{\text{reg},\text{grav}}$ and $\Phi_{\text{reg},B_{i}}$, and the coupling terms between density and the three components of magnetization $\Phi_{\text{VI},\text{grav}-B_{i}}$. The uncertainties of gravity and magnetic data are applied in the data covariance matrix of the misfit terms. Regularization minimizes the difference in density and magnetization gradient between adjacent cells and thus favors a model with smooth variations. The coupling term favors a model with a density that is correlated with each component of magnetization. The objective function reads as

$$\begin{array}{c} \Phi =\Phi_{d,\text{grav}}+\lambda_{\text{reg},\text{grav}}\Phi_{\text{reg},\text{grav}}+\sum_{i=x,y,z}\Phi_{d,B_{i}}+\sum_{i=x,y,z}\lambda_{\text{reg},B}\Phi_{\text{reg},B_{i}}\\ +\sum_{i=x,y,z}\lambda_{\text{VI}}\Phi_{\text{VI},\text{grav}-B_{i}} \end{array}$$

where λ are the weighting terms. For details of this approach, see (*55*, *58*).

We perform the inversion starting with a large coupling weight of $\lambda_{\text{VI}}=10^{6}$ to ensure that the inversion initially enforces strong coupling and provides models with collocated density anomaly and magnetization. The model updates iteratively until the misfit reaches a plateau, then we lower $\lambda_{\text{VI}}$ by an order of magnitude, which releases the coupling constraint and allows the model to better fit the observations (fig. S2). We repeat this procedure until $\lambda_{\text{VI}}=10^{2}$, at which point the model is smooth due to the effect of the regularization term (fig. S4). The regularization weights of magnetization and density are constants of 10 and 100 for the whole run, respectively. In the main text, we focus only on the resulting model with $\lambda_{\text{VI}}=10^{4}$, which fits the observations well while preserving a high degree of collocation between density and magnetization where collocation is suggested by observations (fig. S2).

The final model consists of a 3D representation of density and three vector components of magnetization (fig. S3). We mainly focus on total magnetization, ∣M∣, vertical magnetization, $M_{z}$, and horizontal magnetization, $M_{h}=\sqrt{M_{x}^{2}+M_{y}^{2}}$, for interpretation (Fig. 3). The model has a horizontal resolution of 6 km by 6 km and a vertical resolution of 0.5 km, which increases by a factor of 1.05 for each layer with depth up to its total depth of about 60 km. The model has an upward offset of 5 km to include the surface topography; that is, the model space ranges from 5 km above the mean planetary surface to about 55 km depth.

### Synthetic test

We set up synthetic models (fig. S5) to test (i) how well the inversion constrains the placement and extent of a magnetized body, and (ii) how effectively, using the advantages of coupling, it can recover a magnetized body collocated with a density structure. For all cases, one of the prisms is magnetized but has no density anomaly. We create three scenarios where the second magnetized prism has an overlapping (fig. S5A), partially overlapping (fig. S5B), or nonoverlapping (fig. S5C) density anomaly. Random noise of 1 mGal and 0.1 nT is added to the synthetic observations. After predicting data at 6- and 30-km altitudes, we perform joint inversions of the synthetic models. We show results corresponding to the coupling weight decreasing to $\lambda_{\text{VI}}=10^{4}$.

We note that the magnetized prism that is not associated with a density structure always corresponds to a magnetized body with a consistent magnetization and depth range (fig. S5, G to I). Then, we compare the geometric extent and placement of high-density and magnetized prisms for the different setups, referred to as overlapping, partially overlapping, and nonoverlapping (fig. S5, D to I). Only the overlapping model results in an anomaly body that can be fully reconstructed with respect to density, magnetization, and depth range (fig. S5, D and G). The partially overlapping model shows an anomaly with an elevated density and reduced magnetization compared to the synthetic setup. The extended depth of the high density model block compared to the magnetization model block is not fully captured. Instead, the inversion places an intermediate extent structure (fig. S5, E and H). The nonoverlapping model shows an anomaly body that extends to the same depth of 16 km as the magnetized prism but has a reduced density within a less defined structure compared to the overlapping and partially overlapping models (fig. S5, F and I).

The results of synthetic tests suggest that the joint inversion approach applied in this study can well resolve the density and magnetization horizontally. Within 10 km depth, the vertical extent of the magnetized body can be reliably resolved. However, the depth of density structure will be driven by magnetization depth, so the vertical resolution needs to be interpreted with caution. This is a typical challenge for potential field modeling. Therefore, we conduct an additional degree-depth investigation to estimate the depth range of the density structure described in the next subsection.

### Degree-depth investigation

We assess the depth range of the density anomaly under the Dewar swirl using a degree-depth relation (*59*). The source depth (*d*) of a mass is connected to the spherical harmonic degree, *n*, for a planet with radius, *R*, and can be simplified to d=R/(n+1) for high degrees. Signals from different depths can be isolated by band filtering. With this relation, the relative strength of gravity anomalies in a filtered map provides visual intuition about their corresponding source depth.

Here, we use a sine-shaped high-pass filter with half-degrees (where the filter has a value of 0.5) of 20 to 160, to remove the signals of deep sources in the Bouguer gravity map (fig. S6). The map filtered by a half-degree of 20 shows a large-scale positive Bouguer gravity surrounding the Dewar swirl; this large-scale signal disappears when the half-degree increases to 60, corresponding to a depth of >∼28 km. This depth matches our estimated Moho relief depth at about 33 to 50 km, as derived using the DSP model (compare Fig. 5D and fig. S6, A to C). The remaining small-scale anomaly associated with the Dewar swirl is prominent and invariant on maps with a half-degree of 60 to 160, corresponding to the depth range from ∼28 to ∼11 km (fig. S6, C to F). These observations suggest that the density structure of the Dewar anomaly has a strong portion at a depth of <11 km, which is consistent with the joint inversion results (Fig. 4A), supporting the validity of our inversion model and the associated interpretations.

### Estimate of minimum Paleointensity

We develop a four-step parametric investigation to estimate the minimum paleointensity using our inversion results (fig. S8), in which we (i) constrain the intrusive basalt fraction, (ii) constrain the metallic iron fraction, (iii) derive the reduced ilmenite fraction, and (iv) estimate the minimum paleointensity. The values of the parameters and the corresponding references used are summarized in Table 1.

**Table 1.** Parameters of estimating the minimum paleointensity. Reference values are listed in the table with uncertainties given in the footnote.

Symbol	Description	Value	Reference
$\rho_{c}$	Crustal bulk density	2550 kg/m^3^	(*54*)
$\rho_{b}$	Grain density of basalt	3350 kg/m^3^	(*77*)
*s*	Squareness ratio of the hysteresis loop	0.006*	(*78*)
$M_{c}^{\text{Fe}}$	Saturation magnetization of iron	1.715 × 10^6^ A/m	(*80*)
*a*	Experimental constant	3000 μT†	(*79*)
$c_{\text{il}}$	Volumetric fraction of ilmenite in basalt	2.7%‡	(*81*)
$\rho_{\text{Fe}}$	Grain density of iron	7600 kg/m^3^	
$\rho_{\text{il}}$	Grain density of ilmenite	4800 kg/m^3^	

* 0.004 to 0.008 in (*78*).

† 2810 to 3770 μT in (*1*).

‡ 0.4 to 4.0% in (*81*).

#### Step 1: Constraint on the fraction of basalt

Given that the density anomaly (Δρ), as estimated by our joint inversion, has a magmatic origin, we assume that it originates only from intrusive basalt and

$$\Delta \rho =c_{b}\left[\left(1-\Phi_{b}\right)\rho_{b}-\rho_{c}\right]$$ (1)

where $c_{b}$ is the volumetric fraction of basalt, $\Phi_{b}$ is the porosity of the basalt, $\rho_{b}$ is the grain density of the basalt, and $\rho_{c}$ is the bulk density of the crust. The volumetric fraction of basalt must not be less than $\Delta \rho /\left(P_{b}-\rho_{c}\right)$ or exceed 100%, corresponding to the porosity of the basalt ranging from $0\;\text{to}\;1-\left(\Delta \rho +\rho_{c}\right)/P_{b}$. For consistency, we use the same crustal density as for the DSP model ($\rho_{c}=2550\;\text{kg}/m^{3}$). The grain density of the basalt is assumed to be $\rho_{b}=3350\;\text{kg}/m^{3}$ using the typical value of the low-Ti basalt (*77*).

#### Step 2: Constraint on the fraction of iron

We follow the method from (*24*) to constrain the volumetric fraction of metallic iron ($c_{\text{Fe}}$) using the inverted crustal magnetization anomaly (ΔM)

$$\Delta M=c_{\text{Fe}}{\mathrm{sa}}^{-1}|B|M_{c}^{\text{Fe}}$$ (2)

where *s* is the squareness ratio of the hysteresis loop (*78*), *a* is an experimental constant (*1*, *79*), and $M_{c}^{\text{Fe}}$ is the saturation magnetization of metallic iron (*80*). The parameter ∣B∣ is the paleointensity, that is, the magnetic field active at the time the material was emplaced and magnetized. The magnetic properties of the intrusive basalt are assumed to be similar to those of the Apollo samples of low-Ti basalt.

#### Step 3: Fraction of reduced ilmenite

We assume that all metallic iron is generated from the subsolidus reduction of ilmenite during the slow cooling of magma (*34*, *62*)

$${\text{FeTiO}}_{3}=\text{Fe}+{\text{TiO}}_{2}+\frac{1}{2}O_{2}$$

The fraction of reduced ilmenite, *f*, following this reaction can be derived from the mass ratio of iron to ilmenite

$$f=\gamma^{-1}\frac{\rho_{\text{Fe}}}{\left(1-\Phi_{b}\right)\rho_{\text{il}}}\frac{c_{\text{Fe}}}{c_{b}c_{\text{il}}}$$ (3)

where $\rho_{\text{Fe}}$ is the grain density of iron, $\rho_{\text{il}}$ is the grain density of ilmenite, $c_{\text{il}}$ is the ilmenite concentration in the low-Ti lunar basalt, and γ = 0.368 is the ratio of iron to ilmenite molecular mass of the reduction.

We assume an ilmenite concentration of $c_{\text{il}}=2.7\%$ using the average value of pigeonite basalt from Apollo 12 samples (*81*). The mineralogy of the low-Ti basalt is chosen based on the maximum surface concentration of FeO (about 15 wt%) and TiO_2_ (about 1.5 wt%) shown by the Clementine UVVIS dataset (Fig. 5, A and B) and the high concentration of pyroxene and the low concentration of olivine observed from the Kaguya Multiband Imager dataset [see the global composition map (*82*)].

Here, ilmenite is assumed to have the same porosity as basalt. Combining Eqs. 1 to 3, the fraction of reduced ilmenite is given by

$$f=\gamma^{-1}\frac{\rho_{\text{Fe}}\rho_{b}}{c_{\text{il}}\rho_{\text{il}}}\frac{a}{{\mathrm{sM}}_{c}^{\text{Fe}}|B|}\frac{\Delta M}{\Delta \rho +c_{b}\rho_{c}}$$ (4)

We use the average values of the parameters in Table 1, porosity $\Phi_{b}=12\%$, and a paleointensity of 70 μT, corresponding to the average value during the strong field epoch before ∼3.8 Ga (see Fig. 1). The derived fraction of reduced ilmenite is f≈40% using the maximum depth averaged value of ΔM=0.2 A/m and $\Delta \rho =150\;\text{kg}/m^{3}$ from our inversion results (Fig. 4, C and D).

#### Step 4: Minimum paleointensity

On the basis of Eq. 4, we can further estimate the minimum paleointensity required to generate the magnetic anomaly. Assuming maximum iron availability during the formation of the anomaly, that is, the entire volume consists of basalt ($c_{b}=1$) and all ilmenite is fully reduced (*f* = 1), the magnetic intensity required to generate the observed magnetization ΔM is minimized. Therefore, the minimum paleointensity of the observed anomaly, ${|B|}_{\text{min}}$, is given by

$${|B|}_{\text{min}}=\gamma^{-1}\frac{\rho_{\text{Fe}}\rho_{b}}{c_{\text{il}}\rho_{\text{il}}}\frac{a}{{\mathrm{sM}}_{c}^{\text{Fe}}}\frac{\Delta M}{\Delta \rho +\rho_{c}}$$ (5)

We note that the minimum paleointensity is proportional to the crustal magnetization per mass $\Delta M/\left(\Delta \rho +\rho_{c}\right)$. On the basis of the average magnetization per mass of 73.1 μA m^2^/kg from the joint inversion (Fig. 4E), the estimated minimum paleointensity is ${|B|}_{\text{min}}\approx 11.4\;\text{μT}$. This value is affected by the uncertainties of the parameters and processes involved. In particular, the squareness ratio of the hysteresis loop (*s*) could vary by about 33%. The uncertainty of constant *a* may decrease the estimate by less than 10% or increase it by about 26%. The highest feasible concentration of ilmenite in the pigeonite basalt is about 4%, which decreases the estimate by approximately 33%. In summary, uncertainties could reduce the estimate to about 5.4 μT, still requiring a dynamo field, or increase the estimate by an order of magnitude.

## Supplementary Materials

### This PDF file includes:

Figs. S1 to S8 References
