# Supplementary material for: Magmatic origin of the Dewar magnetic anomaly: Implications for an early lunar dynamo
Source: Sci Adv. 2026 Sep 23;12(39):eaec0341. doi: 10.1126/sciadv.aec0341 (PMC13600156; doi:10.1126/sciadv.aec0341)
Supplement: Supplementary file 1 [file sciadv.aec0341_sm.pdf]

Science Advances
AAAS

Supplementary Materials for

Magmatic origin of the Dewar magnetic anomaly: Implications for an early lunar dynamo

Xi Yang *et al.*

Corresponding author: Xi Yang, xi.yang@eaps.ethz.ch

Sci. Adv. **12**, eaec0341 (2026)
DOI: 10.1126/sciadv.aec0341

This PDF file includes:

Figs. S1 to S8
References

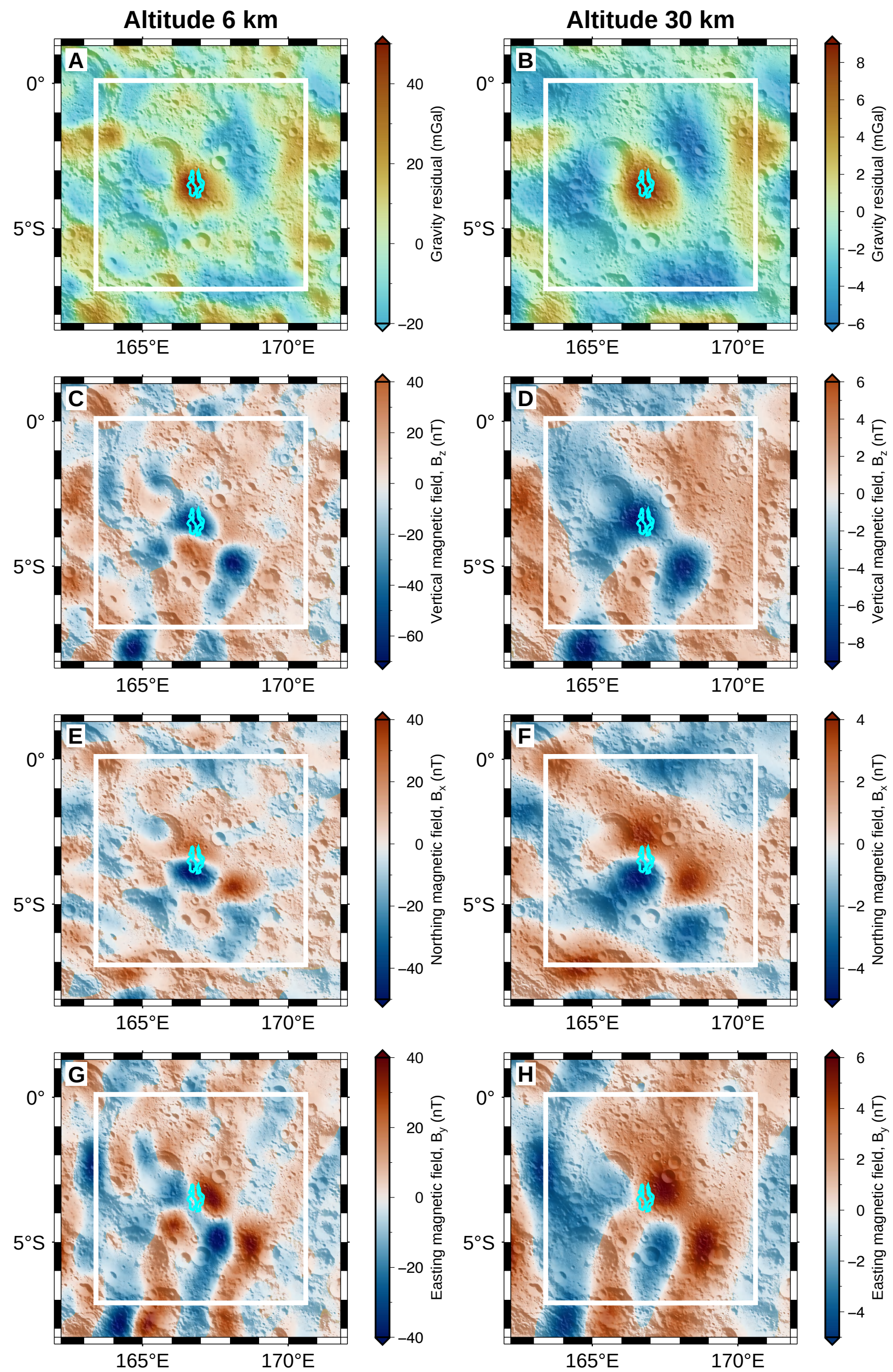

Figure S1: **Gravity and magnetic data in the Dewar region.** (**A,B**) Gravity residuals, (**C,D**) vertical (B_z), (**E,F**) northing (B_x), and (**G,H**) and easting (B_y) component a the magnetic field model (*27*) at the altitudes of 6 km (left column) and 30 km (right column), respectively. These serve as input for the inversion. The boxes denote the model region in Fig. 3 and Fig. S3. The Dewar swirl is highlighted in cyan.

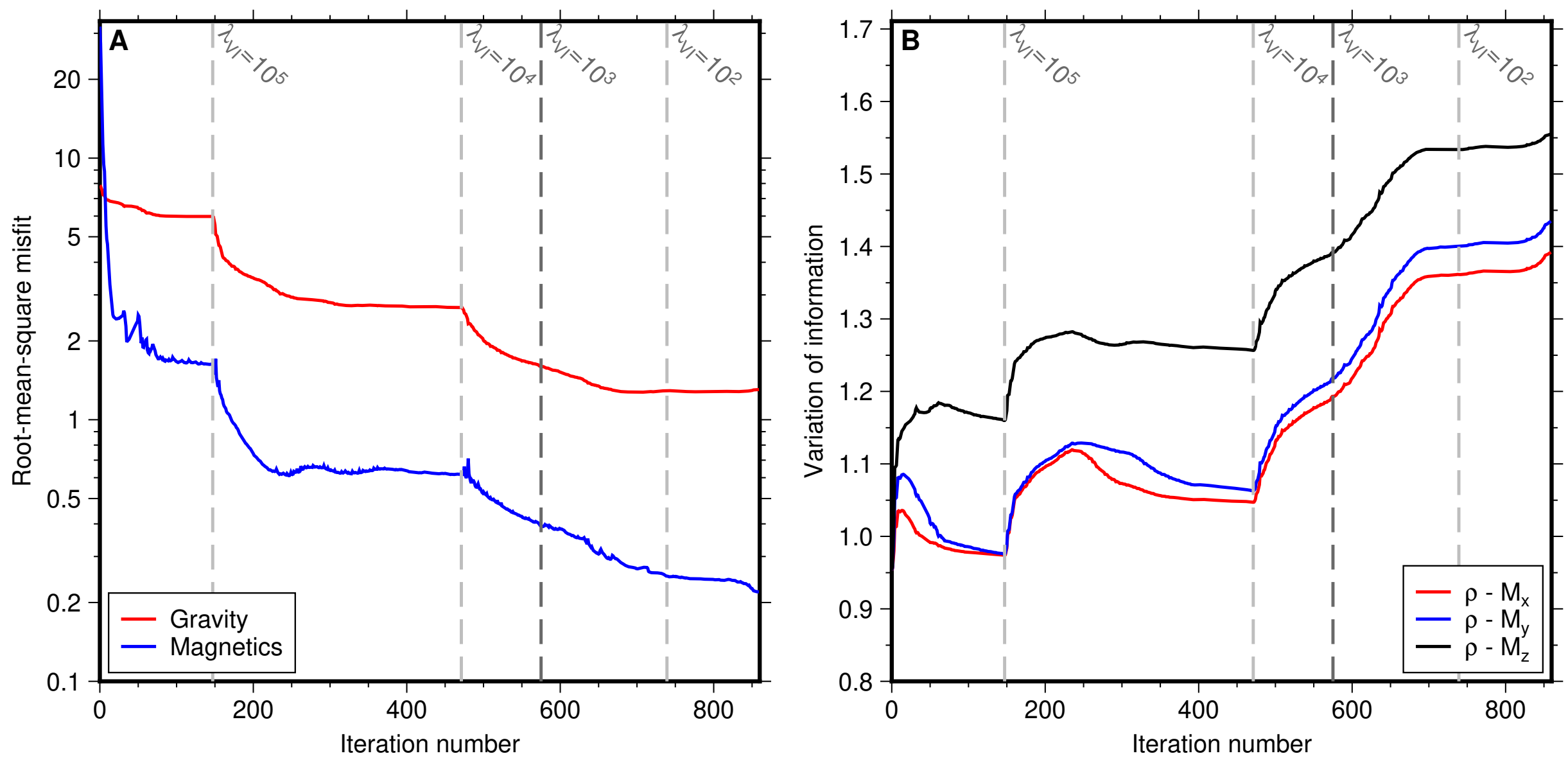

Figure S2: **Misfit and coupling of iterative inversion.** (**A**) The root-mean-square misfits and (**B**) the variation of information coupling are shown as a function of iterations. The vertical gray dashed lines denote the start of new iterations in which the coupling weight is reduced by an order of magnitude. The final results with a coupling weight of 10^4, i.e., the interpreted model solution, are highlighted in dark gray.

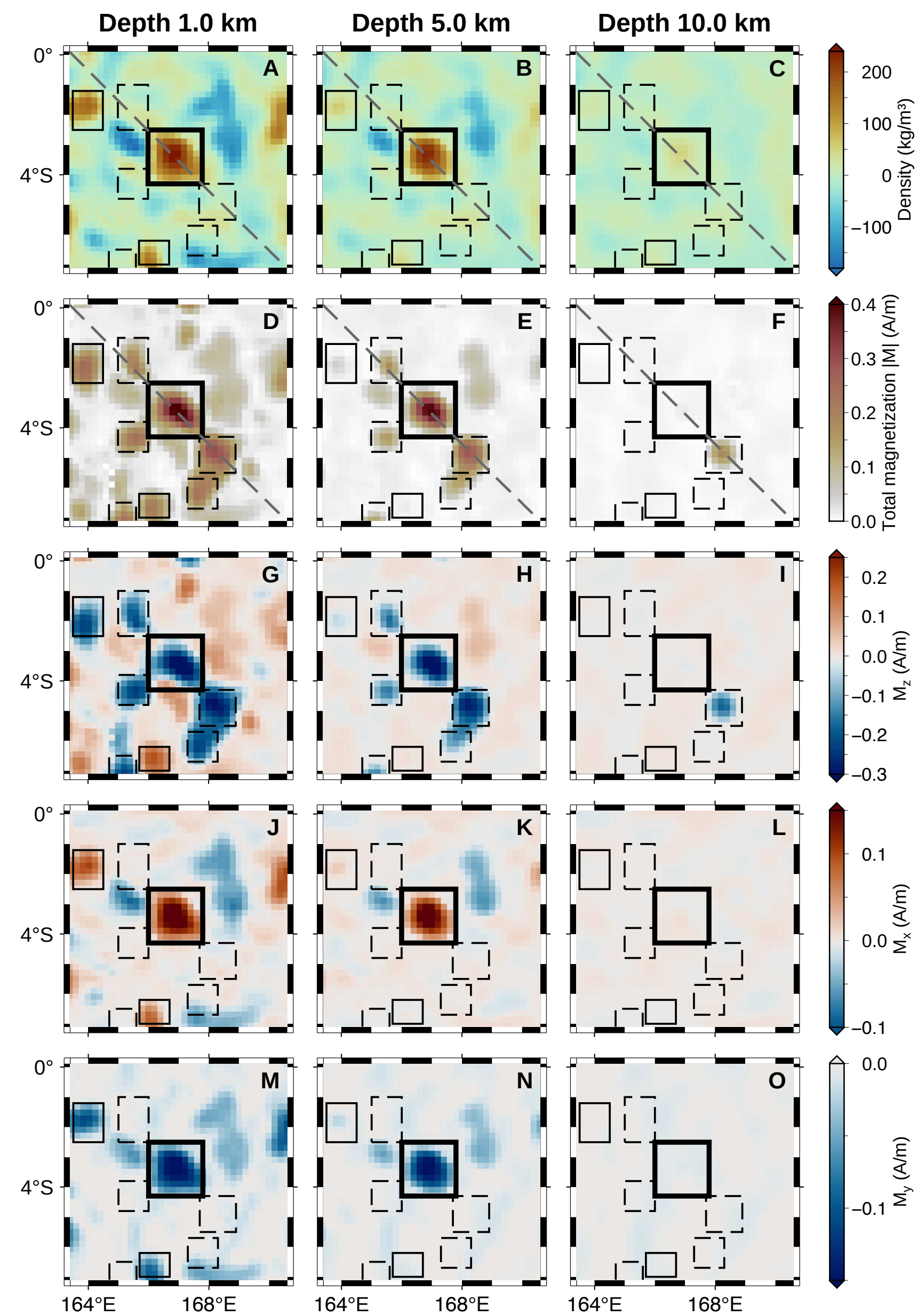

Figure S3: **Crustal structures in the Dewar region.** (**A–C**) Density anomaly, (**D–F**) total magnetization($|M|$), (**G–I**) vertical (M_z), (**J–L**) northing (M_x), and (**M–O**) easting (M_y) components of magnetization of the presented model. From left to right, each column corresponds to a depth slice at 1, 5, and 10 km. The solid and dashed boxes denote magnetized bodies with a positive and no density anomaly, respectively. The gray dashed lines in the first two rows denote the locations of vertical profiles shown in Figs. 4 and S4A,B.

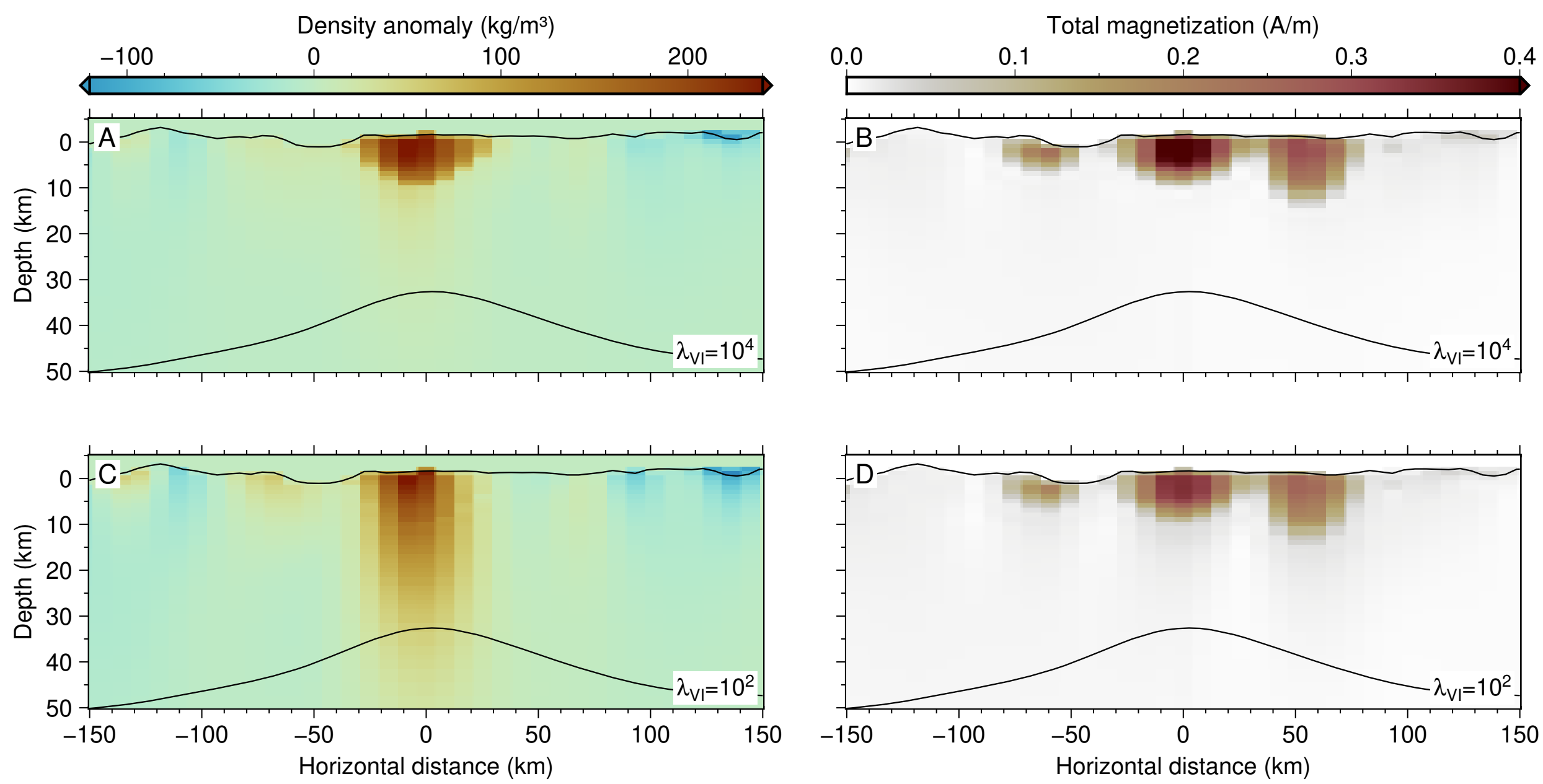

Figure S4: **Structure of the Dewar anomaly.** Vertical profiles of the (**A,C**) density anomaly and (**B,D**) total magnetization. Representative models from a joint inversion with a coupling weight of (A,B) 10^4 and (C,D) 10^2. The solid curves show the surface and Moho relief.

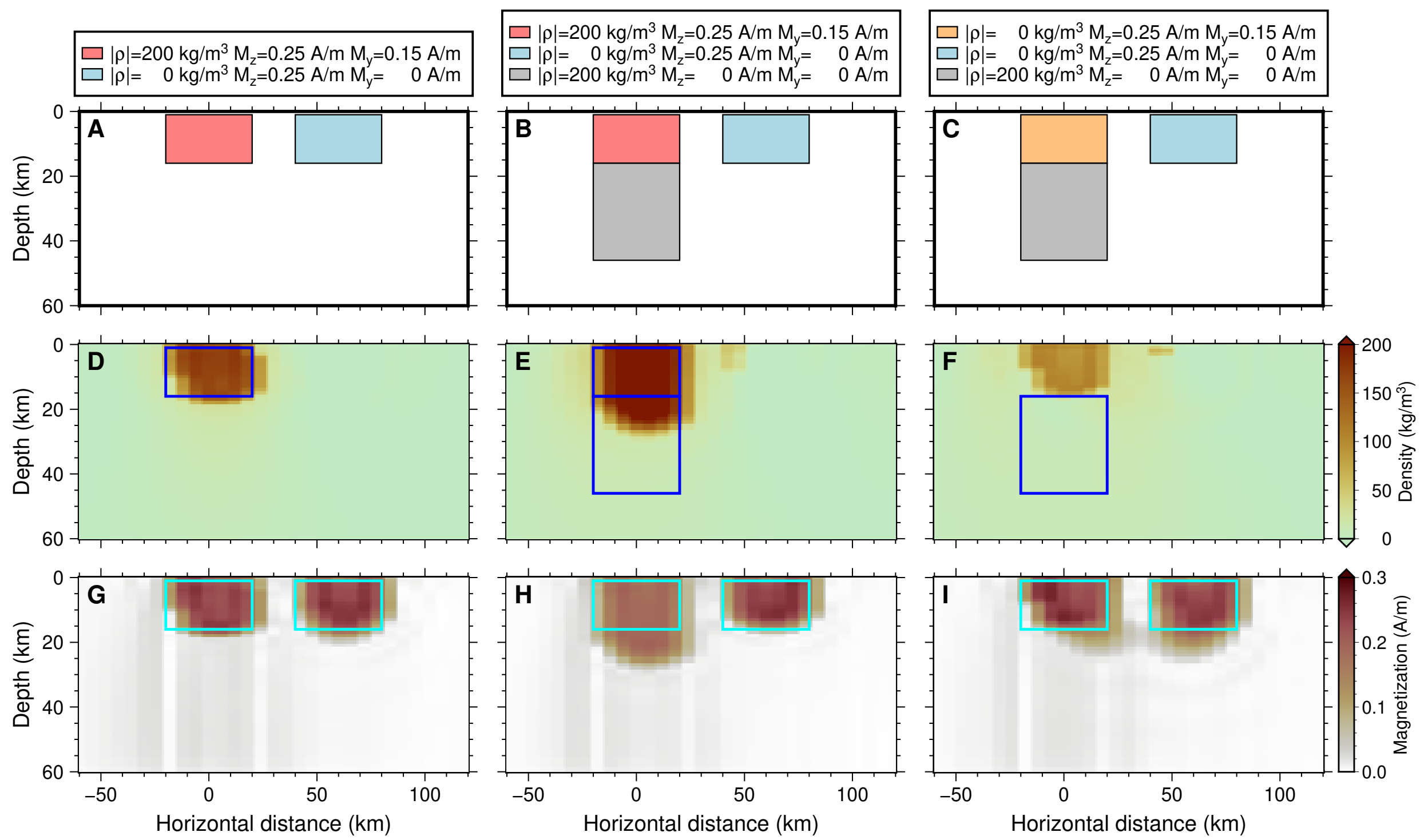

Figure S5: **Summary of synthetic tests.** (**A–C**) the model setups, (**D–F**) the modeled density, and (**G–I**) total magnetization. From left to right, the columns correspond to the overlapping, partially overlapping, and non-overlapping models. (**D–I**) Boxes highlight the locations of anomalies.

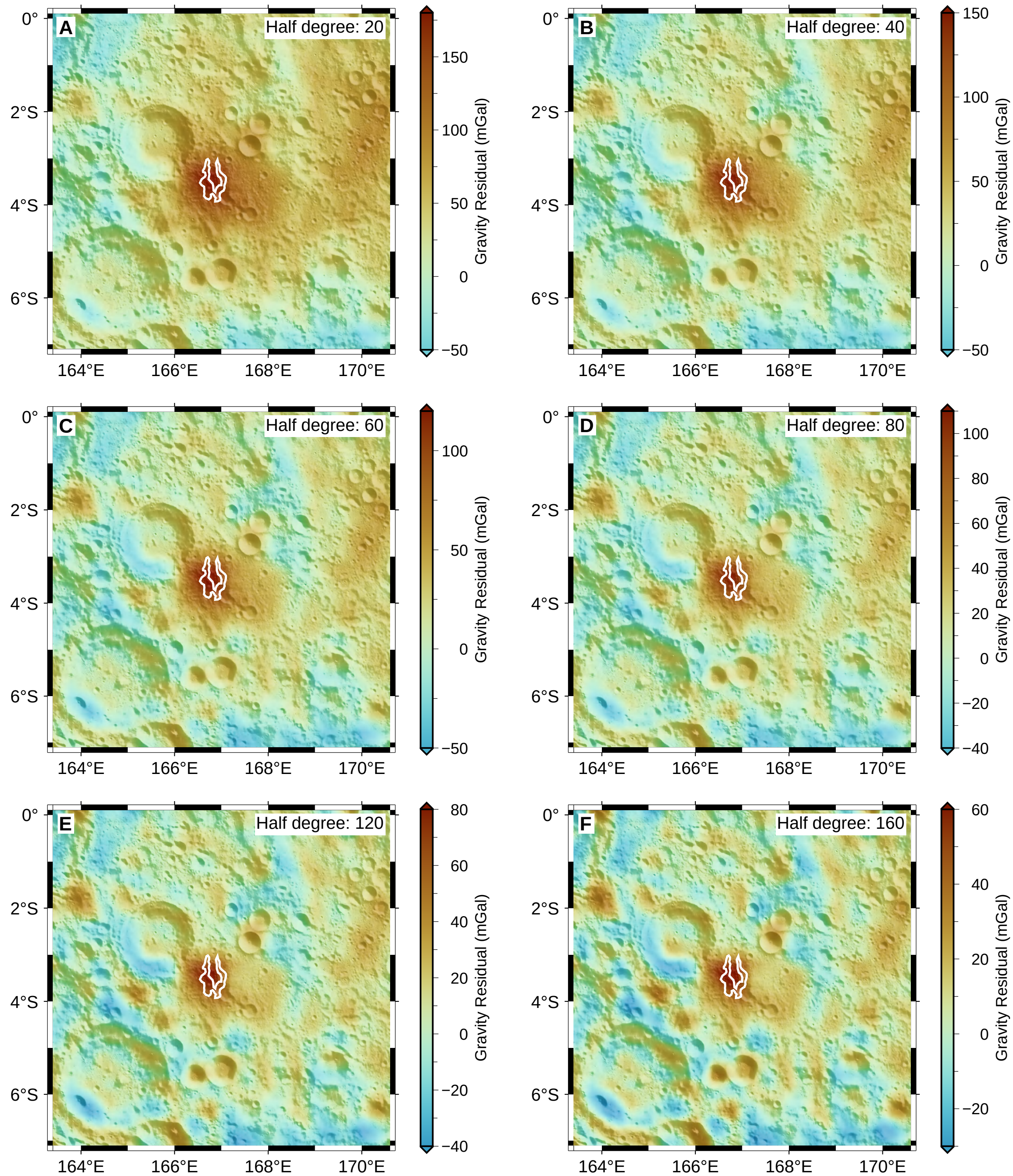

Figure S6: **Filtered Bouguer gravity anomaly map at the surface.** Gravity filtered by a sine-shaped filter with half degrees of (**A**) 20, (**B**) 40, (**C**) 60, (**D**) 80, (**E**) 120, and (**F**) 160. Based on the degree-depth relation (*61*), corresponding source depths are less than about 83, 42, 28, 21, 14, and 11 km, respectively. The Dewar swirl is highlighted in white.

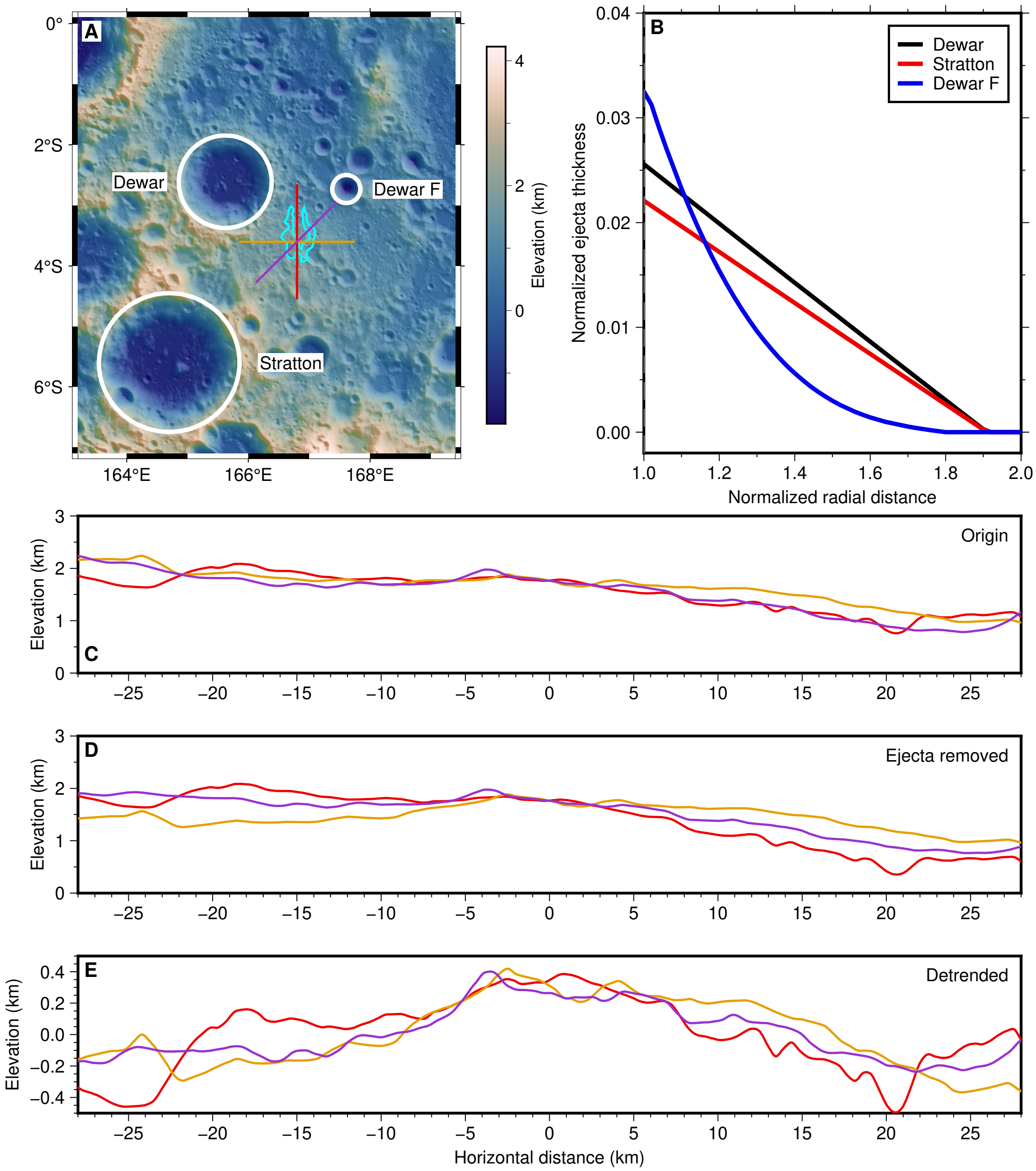

Figure S7: **Topographic profiles of the Dewar swirl.** (**A**) Topographic map with white circles showing the crater rims of Dewar, Stratton, and Dewar F craters. The colored lines show transects across the Dewar swirl (cyan) corresponding to (C–E). (**B**) The ejecta thickness of these craters is derived using the crater model of (*88*). (**C–E**) Profiles in (A) from south or west showing (**C**) the original topographic profiles, (**D**) the profiles processed by removing the ejecta thicknesses from (B), and (**E**) the detrended profiles, which remove the linear trend from (D).

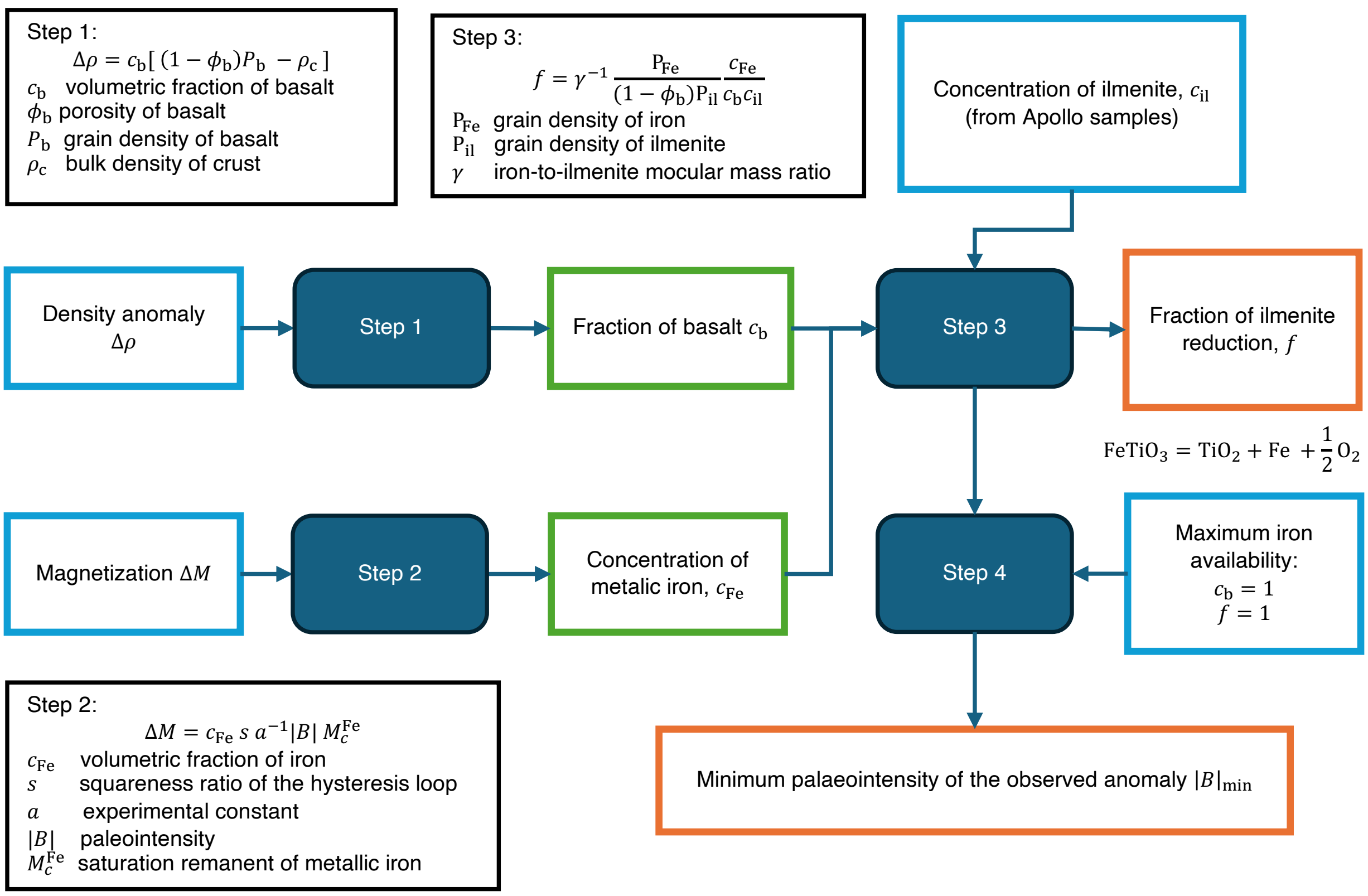

Figure S8: **Flow chart to estimate minimum paleointensity. Step 1:** Estimate the volumetric fraction of basalt based on the density anomaly. **Step 2:** Estimate the volumetric fraction of metallic iron based on the magnetization (*24*). **Step 3:** Estimate the fraction of reduced ilmenite based on the mass relation of the reduction reaction. **Step 4:** Estimate the minimum paleointensity of the observed anomaly by assuming a maximum available amount of iron. We used the concentration of ilmenite in the lunar basalts from the measurements of Apollo samples. Note that the ilmenite is assumed to have the same porosity as the crust. For more details on parameters, see Table 1.

REFERENCES

1. B. P. Weiss, S. M. Tikoo, The lunar dynamo. *Science* **346**, 1246753 (2014).

2. M. A. Wieczorek, B. P. Weiss, D. Breuer, D. Cébron, M. Fuller, I. Garrick-Bethell, J. Gattacceca, J. S. Halekas, D. J. Hemingway, L. L. Hood, M. Laneuville, F. Nimmo, R. Oran, M. E. Purucker, T. Rückriemen, K. M. Soderlund, S. M. Tikoo, Lunar magnetism. *Rev. Mineral. Geochem.* **89**, 207–241 (2023).

3. C. M. Pieters, S. K. Noble, Space weathering on airless bodies. *J. Geophys. Res.: Planets* **121**, 1865–1884 (2016).

4. I. Garrick-Bethell, B. P. Weiss, D. L. Shuster, J. Buz, Early lunar magnetism. *Science* **323**, 356–359 (2009).

5. I. Garrick-Bethell, B. P. Weiss, D. L. Shuster, S. M. Tikoo, M. M. Tremblay, Further evidence for early lunar magnetism from troctolite 76535. *J. Geophys. Res.: Planets* **122**, 76–93 (2017).

6. B. E. Strauss, S. M. Tikoo, J. Gross, J. B. Setera, B. Turrin, Constraining the decline of the lunar dynamo field at ≈3.1 Ga through paleomagnetic analyses of Apollo 12 mare basalts. *J. Geophys. Res.: Planets* **126**, e2020JE006715 (2021).

7. J.-I. Jung, S. M. Tikoo, D. Burns, Z. Váci, M. J. Krawczynski, Assessing lunar paleointensity variability during the 3.9–3.5 Ga high field epoch. *Earth Planet. Sci. Lett.* **638**, 118757 (2024).

8. C. Cournède, J. Gattacceca, P. Rochette, Magnetic study of large Apollo samples: Possible evidence for an ancient centered dipolar field on the Moon. *Earth Planet. Sci. Lett.* **331-332**, 31–42 (2012).

9. C. I. O. Nichols, B. P. Weiss, B. L. Getzin, H. H. Schmitt, A. Béguin, A. S. P. Rae, J. Shah, The palaeoinclination of the ancient lunar magnetic field from an Apollo 17 basalt. *Nat. Astron.* **5**, 1216–1223 (2021).

10. E. K. Shea, B. P. Weiss, W. S. Cassata, D. L. Shuster, S. M. Tikoo, J. Gattacceca, T. L. Grove, M. D. Fuller, A long-lived lunar core dynamo. *Science* **335**, 453–456 (2012).

11. S. M. Tikoo, B. P. Weiss, W. S. Cassata, D. L. Shuster, J. Gattacceca, E. A. Lima, C. Suavet, F. Nimmo, M. D. Fuller, Decline of the lunar core dynamo. *Earth Planet. Sci. Lett.* **404**, 89–97 (2014).

12. K. Lawrence, C. Johnson, L. Tauxe, J. Gee, Lunar paleointensity measurements: Implications for lunar magnetic evolution. *Phys. Earth Planet. Inter.* **168**, 71–87 (2008).

13. J. Buz, B. P. Weiss, S. M. Tikoo, D. L. Shuster, J. Gattacceca, T. L. Grove, Magnetism of a very young lunar glass. *J. Geophys. Res.: Planets* **120**, 1720–1735 (2015).

14. S. M. Tikoo, B. P. Weiss, J. Buz, E. A. Lima, E. K. Shea, G. Melo, T. L. Grove, Magnetic fidelity of lunar samples and implications for an ancient core dynamo. *Earth Planet. Sci. Lett.* **337-338**, 93–103 (2012).

15. S. M. Tikoo, B. P. Weiss, D. L. Shuster, C. Suavet, H. Wang, T. L. Grove, A two-billion-year history for the lunar dynamo. *Sci. Adv.* **3**, e1700207 (2017).

16. S. Cai, K. Qi, S. Yang, J. Fang, P. Shi, Z. Shen, M. Zhang, H. Qin, C. Zhang, X. Li, F. Chen, Y. Chen, J. Li, H. He, C. Deng, C. Li, Y. Pan, R. Zhu, A reinforced lunar dynamo recorded by Chang'e-6 farside basalt. *Nature* **643**, 361–365 (2025).

17. S. Cai, H. Qin, H. Wang, C. Deng, S. Yang, Y. Xu, C. Zhang, X. Tang, L. Gu, X. Li, Z. Shen, M. Zhang, K. He, K. Qi, Y. Fan, L. Dong, Y. Hou, P. Shi, S. Liu, F. Su, Y. Chen, Q. Li, J. Li, R. N. Mitchell, H. He, C. Li, Y. Pan, R. Zhu, Persistent but weak magnetic field at the Moon's midstage revealed by Chang'e-5 basalt. *Sci. Adv.* **11**, eadp3333 (2025).

18. S. Mighani, H. Wang, D. L. Shuster, C. S. Borlina, C. I. O. Nichols, B. P. Weiss, The end of the lunar dynamo. *Sci. Adv.* **6**, eaax0883 (2020).

19. J. A. Tarduno, R. D. Cottrell, K. Lawrence, R. K. Bono, W. Huang, C. L. Johnson, E. G. Blackman, A. V. Smirnov, M. Nakajima, C. R. Neal, T. Zhou, M. Ibanez-Mejia, H. Oda, B. Crummins, Absence of a long-lived lunar paleomagnetosphere. *Sci. Adv.* **7**, eabi7647 (2021).

20. T. Zhou, J. A. Tarduno, R. D. Cottrell, C. R. Neal, F. Nimmo, E. G. Blackman, M. Ibañez-Mejia, A lunar core dynamo limited to the Moon's first 140 million years. *Commun. Earth Environ.* **5**, 456 (2024).

21. D. A. Crawford, Simulations of magnetic fields produced by asteroid impact: Possible implications for planetary paleomagnetism. *Int. J. Impact Eng.* **137**, 103464 (2020).

22. J. S. Halekas, R. P. Lin, D. L. Mitchell, Magnetic fields of lunar multi-ring impact basins. *Meteorit. Planet. Sci.* **38**, 565–578 (2003).

23. L. L. Hood, Central magnetic anomalies of Nectarian-aged lunar impact basins: Probable evidence for an early core dynamo. *Icarus* **211**, 1109–1128 (2011).

24. J. S. Oliveira, M. A. Wieczorek, G. Kletetschka, Iron abundances in lunar impact basin melt sheets from orbital magnetic field data. *J. Geophys. Res.: Planets* **122**, 2429–2444 (2017).

25. X. Yang, M. Wieczorek, Magnetic signatures of lunar impact craters. *Icarus* **415**, 116049 (2024).

26. J. C. Andrews-Hanna, R. C. Weber, I. Garrick-Bethell, A. J. Evans, W. S. Kiefer, R. E. Grimm, J. T. Keane, M. Laneuville, Y. Ishihara, S. Kamata, I. Matsuyama, The structure and evolution of the lunar interior. *Rev. Mineral. Geochem.* **89**, 243–292 (2023).

27. H. Tsunakawa, F. Takahashi, H. Shimizu, H. Shibuya, M. Matsushima, Surface vector mapping of magnetic anomalies over the Moon using Kaguya and Lunar Prospector observations. *J. Geophys. Res.: Planets* **120**, 1160–1185 (2015).

28. D. Ravat, M. E. Purucker, N. Olsen, Lunar magnetic field models from lunar prospector and SELENE/Kaguya along-track magnetic field gradients. *J. Geophys. Res.: Planets* **125**, e2019JE006187 (2020).

29. L. L. Hood, C. B. Torres, J. S. Oliveira, M. A. Wieczorek, S. T. Stewart, A new large-scale map of the lunar crustal magnetic field and its interpretation. *J. Geophys. Res.: Planets* **126**, e2020JE006667 (2021).

30. L. L. Hood, I. Bryant, J. Van Der Leeuw, Lunar magnetic anomalies and polar ice. *Geophys. Res. Lett.* **49**, e2022GL100557 (2022).

31. M. A. Wieczorek, B. P. Weiss, S. T. Stewart, An impactor origin for lunar magnetic anomalies. *Science* **335**, 1212–1215 (2012).

32. L. L. Hood, N. A. Artemieva, Antipodal effects of lunar basin-forming impacts: Initial 3D simulations and comparisons with observations. *Icarus* **193**, 485–502 (2008).

33. I. S. Narrett, R. Oran, Y. Chen, K. Miljković, G. Tóth, E. N. Mansbach, B. P. Weiss, Impact plasma amplification of the ancient lunar dynamo. *Sci. Adv.* **11**, eadr7401 (2025).

34. D. J. Hemingway, S. M. Tikoo, Lunar swirl morphology constrains the geometry, magnetization, and origins of lunar magnetic anomalies. *J. Geophys. Res.: Planets* **123**, 2223–2241 (2018).

35. B. W. Denevi, M. S. Robinson, A. K. Boyd, D. T. Blewett, R. L. Klima, The distribution and extent of lunar swirls. *Icarus* **273**, 53–67 (2016).

36. L. L. Hood, G. Schubert, Lunar magnetic anomalies and surface optical properties. *Science* **208**, 49–51 (1980).

37. R. A. Bamford, E. P. Alves, F. Cruz, B. J. Kellett, R. A. Fonseca, L. O. Silva, R. M. G. M. Trines, J. S. Halekas, G. Kramer, E. Harnett, R. A. Cairns, R. Bingham, 3D PIC simulations of collisionless shocks at lunar magnetic anomalies and their role in forming lunar swirls. *Astrophys. J.* **830**, 146 (2016).

38. J. Deca, A. Divin, C. Lue, T. Ahmadi, M. Horányi, Reiner Gamma albedo features reproduced by modeling solar wind standoff. *Commun. Phys.* **1**, 12 (2018).

39. J. Deca, D. J. Hemingway, A. Divin, C. Lue, A. R. Poppe, I. Garrick-Bethell, B. Lembège, M. Horányi, Simulating the Reiner Gamma swirl: The long-term effect of solar wind standoff. *J. Geophys. Res.: Planets* **125**, e2019JE006219 (2020).

40. I. Garrick-Bethell, J. W. Head, C. M. Pieters, Spectral properties, magnetic fields, and dust transport at lunar swirls. *Icarus* **212**, 480–492 (2011).

41. C. M. Pieters, D. P. Moriarty, I. Garrick-Bethell, "Atypical regolith processes hold the key to enigmatic lunar swirls," in *45th Annual Lunar and Planetary Science Conference* (2014), p. 1408.

42. D. Domingue, J. Weirich, F. Chuang, A. Sickafoose, E. Palmer, Topographic correlations within lunar swirls in Mare Ingenii. *Geophys. Res. Lett.* **49**, e2021GL095285 (2022).

43. D. Hemingway, I. Garrick-Bethell, Magnetic field direction and lunar swirl morphology: Insights from Airy and Reiner Gamma. *J. Geophys. Res.: Planets* **117**, 2012JE004165 (2012).

44. I. Garrick-Bethell, M. R. Kelley, Reiner Gamma: A Magnetized Elliptical Disk on the Moon. *Geophys. Res. Lett.* **46**, 5065–5074 (2019).

45. J. S. Oliveira, M. A. Wieczorek, Testing the axial dipole hypothesis for the Moon by modeling the direction of crustal magnetization. *J. Geophys. Res.: Planets* **122**, 383–399 (2017).

46. J. S. Oliveira, F. Vervelidou, M. A. Wieczorek, M. Díaz Michelena, Constraints on the spatial distribution of lunar crustal magnetic sources from orbital magnetic field data. *J. Geophys. Res.: Planets* **129**, e2023JE008125 (2024).

47. J. C. Andrews-Hanna, S. W. Asmar, J. W. Head III, W. S. Kiefer, A. S. Konopliv, F. G. Lemoine, I. Matsuyama, E. Mazarico, P. J. McGovern, H. J. Melosh, G. A. Neumann, F. Nimmo, R. J. Phillips, D. E. Smith, S. C. Solomon, G. J. Taylor, M. A. Wieczorek, J. G. Williams, M. T. Zuber, Ancient igneous intrusions and early expansion of the moon revealed by GRAIL gravity gradiometry. *Science* **339**, 675–678 (2013).

48. M. E. Purucker, J. W. Head, L. Wilson, Magnetic signature of the lunar South Pole-Aitken basin: Character, origin, and age. *J. Geophys. Res.: Planets* **117**, 2011JE003922 (2012).

49. S. Gong, M. A. Wieczorek, Is the lunar magnetic field correlated with gravity or topography? *J. Geophys. Res.: Planets* **125**, e2019JE006274 (2020).

50. S. J. Lawrence, B. R. Hawke, J. J. Gillis-Davis, G. J. Taylor, D. J. Lawrence, J. T. Cahill, J. J. Hagerty, P. G. Lucey, G. A. Smith, K. Keil, Composition and origin of the Dewar geochemical anomaly. *J. Geophys. Res.: Planets* **113**, 2007JE002904 (2008).

51. J. Whitten, J. W. Head, Lunar cryptomaria: Mineralogy and composition of ancient volcanic deposits. *Planet. Space Sci.* **106**, 67–81 (2015).

52. S. Goossens, T. J. Sabaka, M. A. Wieczorek, G. A. Neumann, E. Mazarico, F. G. Lemoine, J. B. Nicholas, D. E. Smith, M. T. Zuber, High-resolution gravity field models from GRAIL data and implications for models of the density structure of the moon's crust. *J. Geophys. Res.: Planets* **125**, e2019JE006086 (2020).

53. A. Broquet, M. A. Wieczorek, W. Fa, The composition of the south polar cap of Mars derived from orbital data. *J. Geophys. Res.: Planets* **126**, e2020JE006730 (2021).

54. M. A. Wieczorek, G. A. Neumann, F. Nimmo, W. S. Kiefer, G. J. Taylor, H. J. Melosh, R. J. Phillips, S. C. Solomon, J. C. Andrews-Hanna, S. W. Asmar, A. S. Konopliv, F. G. Lemoine, D. E. Smith, M. M. Watkins, J. G. Williams, M. T. Zuber, The crust of the moon as seen by GRAIL. *Science* **339**, 671–675 (2013).

55. A. Mittelholz, M. Moorkamp, A. Broquet, L. Ojha, Gravity and magnetic field signatures in hydrothermally affected regions on Mars. *J. Geophys. Res.: Planets* **130**, e2024JE008832 (2025).

56. A. Broquet, J. Maia, M. A. Wieczorek, On the crustal architecture of the terrestrial planets. *J. Geophys. Res.: Planets* **130**, e2025JE009139 (2025).

57. M. Moorkamp, Deciphering the state of the lower crust and upper mantle with multi-physics inversion. *Geophys. Res. Lett.* **49**, e2021GL096336 (2022).

58. M. Moorkamp, "Joint inversion of gravity and magnetotelluric data from the Ernest-Henry IOCG deposit with a variation of information constraint," in *First International Meeting for Applied Geoscience & Energy Expanded Abstracts* (Society of Exploration Geophysicists, 2021), pp. 1711–1715, doi:10.1190/segam2021-3582000.1.

59. S. Goossens, D. E. Smith, Gravity degree-depth relationship using point mass spherical harmonics. *Geophys. J. Int.* **233**, 1878–1889 (2023).

60. L. M. Jozwiak, J. W. Head, M. T. Zuber, D. E. Smith, G. A. Neumann, Lunar floor-fractured craters: Classification, distribution, origin and implications for magmatism and shallow crustal structure. *J. Geophys. Res.: Planets* **117**, 2012JE004134 (2012).

61. G. A. Neumann, M. T. Zuber, M. A. Wieczorek, J. W. Head, D. M. H. Baker, S. C. Solomon, D. E. Smith, F. G. Lemoine, E. Mazarico, T. J. Sabaka, S. J. Goossens, H. J. Melosh, R. J. Phillips, S. W. Asmar, A. S. Konopliv, J. G. Williams, M. M. Sori, J. M. Soderblom, K. Miljković, J. C. Andrews-Hanna, F. Nimmo, W. S. Kiefer, Lunar impact basins revealed by Gravity Recovery and Interior Laboratory measurements. *Sci. Adv.* **1**, e1500852 (2015).

62. Y. Liang, S. M. Tikoo, M. J. Krawczynski, Possibility of lunar crustal magmatism producing strong crustal magnetism. *J. Geophys. Res.: Planets* **129**, e2023JE008179 (2024).

63. S. C. Steele, R. R. Fu, A. Mittelholz, A. I. Ermakov, R. I. Citron, R. J. Lillis, Weak magnetism of Martian impact basins may reflect cooling in a reversing dynamo. *Nat. Commun.* **15**, 6831 (2024).

64. T. Chaffee, S. M. Tikoo, R. E. Maxwell, I. Garrick-Bethell, Testing the fidelity of paleopole determinations from multidirectionally magnetized lunar crustal anomaly source bodies. *J. Geophys. Res.: Planets* **130**, e2024JE008805 (2025).

65. R. I. Citron, D. E. Smith, S. T. Stewart, L. L. Hood, M. T. Zuber, The South Pole-Aitken basin: Constraints on impact excavation, melt, and ejecta. *Geophys. Res. Lett.* **51**, e2024GL110034 (2024).

66. C. Orgel, G. Michael, C. I. Fassett, C. H. van der Bogert, C. Riedel, T. Kneissl, H. Hiesinger, Ancient bombardment of the inner solar system: Reinvestigation of the “fingerprints” of different impactor populations on the lunar surface. *J. Geophys. Res.: Planets* **123**, 748–762 (2018).

67. M. Laneuville, M. A. Wieczorek, D. Breuer, N. Tosi, Asymmetric thermal evolution of the Moon. *J. Geophys. Res.: Planets* **118**, 1435–1452 (2013).

68. A. J. Evans, M. T. Zuber, B. P. Weiss, S. M. Tikoo, A wet, heterogeneous lunar interior: Lower mantle and core dynamo evolution. *J. Geophys. Res.: Planets* **119**, 1061–1077 (2014).

69. M. Laneuville, M. A. Wieczorek, D. Breuer, J. Aubert, G. Morard, T. Rückriemen, A long-lived lunar dynamo powered by core crystallization. *Earth Planet. Sci. Lett.* **401**, 251–260 (2014).

70. A. Scheinberg, K. Soderlund, G. Schubert, Magnetic field generation in the lunar core: The role of inner core growth. *Icarus* **254**, 62–71 (2015).

71. M. Le Bars, M. A. Wieczorek, O. Karatekin, D. Cébron, M. Laneuville, An impact-driven dynamo for the early Moon. *Nature* **479**, 215–218 (2011).

72. C. Stys, M. Dumberry, A past lunar dynamo thermally driven by the precession of its inner core. *J. Geophys. Res.: Planets* **125**, e2020JE006396 (2020).

73. A. L. Scheinberg, K. M. Soderlund, L. T. Elkins-Tanton, A basal magma ocean dynamo to explain the early lunar magnetic field. *Earth Planet. Sci. Lett.* **492**, 144–151 (2018).

74. C. A. Dwyer, D. J. Stevenson, F. Nimmo, A long-lived lunar dynamo driven by continuous mechanical stirring. *Nature* **479**, 212–214 (2011).

75. A. J. Evans, S. M. Tikoo, An episodic high-intensity lunar core dynamo. *Nat. Astron.* **6**, 325–330 (2022).

76. M. Moorkamp, B. Heincke, M. Jegen, A. W. Roberts, R. W. Hobbs, A framework for 3-D joint inversion of MT, gravity and seismic refraction data: 3-D joint inversion. *Geophys. J. Int.* **184**, 477–493 (2011).

77. W. S. Kiefer, R. J. Macke, D. T. Britt, A. J. Irving, G. J. Consolmagno, The density and porosity of lunar rocks. *Geophys. Res. Lett.* **39**, 2012GL051319 (2012).

78. P. Rochette, J. Gattacceca, A. Ivanov, M. Nazarov, N. Bezaeva, Magnetic properties of lunar materials: Meteorites, Luna and Apollo returned samples. *Earth Planet. Sci. Lett.* **292**, 383–391 (2010).

79. J. Gattacceca, P. Rochette, Toward a robust normalized magnetic paleointensity method applied to meteorites. *Earth Planet. Sci. Lett.* **227**, 377–393 (2004).

80. D. Dunlop, Ö. Özdemir, G. Schubert, Magnetizations in rocks and minerals. *Treatise Geophysics* **5**, 277–336 (2007).

81. C. R. Neal, M. D. Hacker, G. A. Snyder, L. A. Taylor, Y. G. Liu, R. A. Schmitt, Basalt generation at the Apollo 12 site, Part 1: New data, classification, and re-evaluation. *Meteoritics* **29**, 334–348 (1994).

82. M. Lemelin, P. G. Lucey, K. Miljković, L. R. Gaddis, T. Hare, M. Ohtake, The compositions of the lunar crust and upper mantle: Spectral analysis of the inner rings of lunar impact basins. *Planet. Space Sci.* **165**, 230–243 (2019).

83. I. Garrick-Bethell, M. T. Zuber, Elliptical structure of the lunar South Pole-Aitken basin. *Icarus* **204**, 399–408 (2009).

84. J. J. Gillis, B. L. Jolliff, R. C. Elphic, A revised algorithm for calculating TiO_2 from Clementine UVVIS data: A synthesis of rock, soil, and remotely sensed TiO_2 concentrations. *J. Geophys. Res.: Planets* **108**, 2001JE001515 (2003).

85. J. J. Gillis, B. L. Jolliff, R. L. Korotev, Lunar surface geochemistry: Global concentrations of Th, K, and FeO as derived from lunar prospector and Clementine data. *Geochim. Cosmochim. Acta* **68**, 3791–3805 (2004).

86. M. A. Wieczorek, M. Meschede, SHTools: Tools for working with spherical harmonics. *Geochem. Geophys. Geosyst.* **19**, 2574–2592 (2018).

87. F. Crameri, G. E. Shephard, P. J. Heron, The misuse of colour in science communication. *Nat. Commun.* **11**, 5444 (2020).

88. J. Du, W. Fa, M. A. Wieczorek, M.-H. Zhu, Thickness of lunar mare basalts: New results based on modeling the degradation of partially buried craters. *J. Geophys. Res.: Planets* **124**, 2430–2459 (2019).
